# Islands of linkage in an ocean of pervasive recombination reveals two-speed evolution of human cytomegalovirus genomes

**DOI:** 10.1093/ve/vew017

**Published:** 2016-06-15

**Authors:** Florent Lassalle, Daniel P. Depledge, Matthew B. Reeves, Amanda C. Brown, Mette T. Christiansen, Helena J. Tutill, Rachel J. Williams, Katja Einer-Jensen, Jolyon Holdstock, Claire Atkinson, Julianne R. Brown, Freek B. van Loenen, Duncan A. Clark, Paul D. Griffiths, Georges M.G.M. Verjans, Martin Schutten, Richard S.B. Milne, Francois Balloux, Judith Breuer

**Affiliations:** ^1^ UCL Genetics Institute, University College London, London, United Kingdom; ^2^ Division of Infection and Immunity, University College London, London, United Kingdom; ^3^ Oxford Gene Technology, Begbroke, Oxfordshire, UK; ^4^ QIAGEN-AAR, Aarhus, Denmark; ^5^ Department of Virology, Royal Free Hospital, London, United Kingdom; ^6^ Microbiology, Virology and Infection Prevention and Control, Camelia Botnar Laboratories, Great Ormond Street Hospital for Children NHS Foundation Trust, London, United Kingdom; ^7^ Department of Viroscience, Erasmus, MC Rotterdam, the Netherlands; ^8^ Department of Virology, Barts Health NHS Trust, London, United Kingdom

**Keywords:** CMV, recombination, immune evasion, viral evolution

## Abstract

Human cytomegalovirus (HCMV) infects most of the population worldwide, persisting throughout the host's life in a latent state with periodic episodes of reactivation. While typically asymptomatic, HCMV can cause fatal disease among congenitally infected infants and immunocompromised patients. These clinical issues are compounded by the emergence of antiviral resistance and the absence of an effective vaccine, the development of which is likely complicated by the numerous immune evasins encoded by HCMV to counter the host's adaptive immune responses, a feature that facilitates frequent super-infections. Understanding the evolutionary dynamics of HCMV is essential for the development of effective new drugs and vaccines. By comparing viral genomes from uncultivated or low-passaged clinical samples of diverse origins, we observe evidence of frequent homologous recombination events, both recent and ancient, and no structure of HCMV genetic diversity at the whole-genome scale. Analysis of individual gene-scale loci reveals a striking dichotomy: while most of the genome is highly conserved, recombines essentially freely and has evolved under purifying selection, 21 genes display extreme diversity, structured into distinct genotypes that do not recombine with each other. Most of these hyper-variable genes encode glycoproteins involved in cell entry or escape of host immunity. Evidence that half of them have diverged through episodes of intense positive selection suggests that rapid evolution of hyper-variable loci is likely driven by interactions with host immunity. It appears that this process is enabled by recombination unlinking hyper-variable loci from strongly constrained neighboring sites. It is conceivable that viral mechanisms facilitating super-infection have evolved to promote recombination between diverged genotypes, allowing the virus to continuously diversify at key loci to escape immune detection, while maintaining a genome optimally adapted to its asymptomatic infectious lifecycle.

## 1. Introduction

Cytomegalovirus (CMV) species are widespread among mammals, establishing life-long infections in their specific host. Human cytomegalovirus (HCMV) has a linear dsDNA genome of approximately 235 kb encoding at least 165 canonical ORFs (Dolan et al. 2004), plus many alternative transcripts and pervasive translation outside these annotated ORFs revealed by recent expression analyses (Murphy et al. 2003; Stern-Ginossar et al. 2012). The genome of HCMV consists of two domains (L and S), each comprising a unique region (U_L_ and U_S_) flanked by an inverted repeat sequence (TR_L_ and IR_L_, TR_S_ and IR_S_), yielding the genome configuration TR_L_–U_L_–IR_L_–IR_S_–U_S_–TR_S_. While primary infection of healthy individuals is common but usually asymptomatic, HCMV infections of severely immune-suppressed individuals (e.g. HIV-infected and transplant patients) cause significant morbidity and mortality (Rubin 1989; Snydman 1990; Krause et al. 2013; Manicklal et al. 2013). Additionally, trans-placental transmissions of HCMV are the commonest viral cause of severe birth defects in infants (Ross et al. 2006). These problems are compounded by growing antiviral resistance and the lack of an effective vaccine (Krause et al. 2013) and there remains an unmet need for data on viral diversity and HCMV evolution to facilitate the development of new intervention strategies to prevent and/or counteract HCMV pathology. Very recently, the high frequency of recombination at the genome-wide level has been observed (Sijmons et al. 2015). This sheds new light on the understanding of HCMV evolution, in particular regarding recent studies where rapid fixation of novel point mutations was thought to be the main mechanism for intra-host diversification (Renzette et al. 2011; Renzette et al. 2013). However, estimation of the local recombination intensity along the genome is subject to biases when other evolutionary factors vary, and methodological issues may account for some patterns reported by Sijmons et al. (2015).

Here, we explored the landscape of recombination across HCMV genomes and its relation to selective constraints. We used a diverse dataset of genomes recovered from unrelated clinical samples collected in the UK, the Netherlands, Germany, Belgium, Italy, the USA and South Korea for which whole genome sequences were previously published (Bradley et al. 2009; Cunningham et al. 2010; Dargan et al. 2010; Jung et al. 2011; Sijmons et al. 2014; Sijmons et al. 2015), in addition to 16 new HCMV genomes sequences obtained directly from clinical samples. Our results confirm that extensive recombination affects HCMV genomes to the extent that the majority of sites in the genome are unlinked. In addition, we show that there are exceptions to this pattern of free recombination within several loci that are deeply divergent between strains and in high linkage disequilibrium (LD). The largest of these linked hypervariable loci spans the RL11 region, which encodes glycoproteins with multiple functions related to immunity and tropism. The rapid evolution of this locus, both within HCMV and between primate CMV species, suggests an important role for these genes in CMV genome adaptation to their host. Taken together, these patterns support the idea of homologous recombination being a major force in the evolution of HCMV.

## 2. Materials and methods

### 2.1. Sample collection and sequencing

Twenty-five samples were collected from various hospitals in London, UK (detailed in Table 1) from a population reflecting the highly heterogeneous ethnic and geographical origin of London's population. Samples were collected from a mixture of congenitally infected infants as well as children and adults with either competent or suppressed immune statuses with varying HCMV loads. The sample collection included two sets of paired samples (whole blood and bile/whole blood and a solid tissue biopsy) from different body compartments and three sets of longitudinally collected samples from either whole blood or plasma (Table 1). DNA was extracted using the QIAamp DNA Mini Kit (QIAGEN) according to the instructions of the manufacturer and quality assured using the Qubit and Tapestation systems. Viral loads were determined for each sample using a diagnostic qPCR assay as follows. A 15 µL mastermix (12.5 µL of QuantiFast Multiplex PCR +R and 2.5 µL of Primer/Probe mix) was combined with 10 µL of extracted DNA. Initial denaturation was at 95 °C for 5 min followed by 45 cycles of denaturation (95 °C for 30 s) and annealing/extension (60 °C for 30 s). Forward (GCATGCGC GAGTGTCAAGAC) and reverse (GTTACTTT GAGCGCCATCTGT TCCT) primers were used at a final reaction concentration of 0.6 pmol/µL while the probe (TGCGCCGTATGCTGCTCGACA, reporter = JOE, quencher = BHQ1) was used at a final concentration of 0.4 pmol/µL. All reactions were run in triplicate on an Applied Biosystems 7500 Fast Real-Time PCR System using six standards ranging from 500 copies/mL to 20,000,000 copies/mL. 

**Table 1.** List of genomes sequences used in the present study and associated metadata

Strain name	GenBank accession	Country of isolation	Sample type	Immune status
NL/Rot1/Urine/2012	KT726940	Netherlands	Urine (1 passage)	Competent
NL/Rot2/Urine/2012	KT726941	Netherlands	Urine (1 passage)	Competent
NL/Rot3/Nasal/2012	KT726942	Netherlands	Nasal rinse (1 passage)	Competent
NL/Rot4/Nasal/2012	KT726943	Netherlands	Nasal rinse (1 passage)	Competent
NL/Rot5/Urine/2012	KT726944	Netherlands	Urine (1 passage)	Competent
NL/Rot6/Nasal/2012	KT726945	Netherlands	Nasal rinse (1 passage)	Competent
NL/Rot7/Urine/2012	KT726946	Netherlands	Urine (1 passage)	Competent
UK/Lon1/Blood/2013	KT726947	UK	Blood (EDTA)^a^	Compromised
UK/Lon2/Blood/2013	KT726948	UK	Blood (EDTA)^a^	Compromised
UK/Lon6/Urine/2011	KT726949	UK	Urine	Competent
UK/Lon7/Urine/2011	KT726950	UK	Urine	Competent
UK/Lon8/Urine/2012	KT726951	UK	Urine	Competent
UK/Lon3/Plasma/2012	KT726952	UK	Plasma^a^	Compromised
UK/Lon9/Urine/2012	KT726953	UK	Urine	Competent
UK/Lon4/Bile/2011	KT726954	UK	Whole blood & bile^b^	Compromised
UK/Lon5/Blood/2010	KT726955	UK	Whole blood & biopsy^b^	Compromised
Merlin	NC_006273	UK	Urine	Competent
3157	GQ221974	UK	Urine (3 passage)	Competent
JP	GQ221975	UK	Biopsy (prostate)	Compromised
HAN20	GQ396663	Germany	BAL (2 pass)	Unknown
HAN38	GQ396662	Germany	BAL (2 pass)	Unknown
3301	GQ466044	UK	Urine	Competent
JHC	HQ380895	South Korea	Whole blood	Compromised
U8	GU179288	Italy	Urine	Competent
U11	GU179290	UK	Urine	Competent
VR1814	GU179289	Italy	Cervical secretion	Competent
TR	KF021605	USA	Vitreuos humor (>4 pass)	Compromised
BE/9/2010	KC519319	Belgium	Urine (2 pass)	Competent
BE/10/2010	KC519320	Belgium	Urine (2 pass)	Competent
BE/11/2010	KC519321	Belgium	Urine (2 pass)	Competent
BE/21/2010	KC519322	Belgium	Urine	Compromised
BE/27/2010	KC519323	Belgium	Urine (4 pass)	Compromised
HAN1	JX512199	Germany	BAL	Unknown
HAN2	JX512200	Germany	BAL (3 pass)	Unknown
HAN3	JX512201	Germany	BAL (3 pass)	Unknown
HAN8	JX512202	Germany	BAL (3 pass)	Unknown
HAN12	JX512203	Germany	BAL (3 pass)	Unknown
HAN16	JX512204	Germany	Urine (2 pass)	Unknown
HAN19	JX512205	Germany	BAL (2 pass)	Unknown
HAN22	JX512206	Germany	BAL (2 pass)	Unknown
HAN28	JX512207	Germany	BAL (3 pass)	Unknown
HAN31	JX512208	Germany	BAL (2 pass)	Unknown

pass, passage.

^a^Longitudinal sampling (3 timepoints).

^b^Simultaneous sampling of two bodycompartments.

The 120-mer RNA baits spanning the length of the positive strand of 15 whole and 44 partial HCMV genome sequences (as obtained from GenBank in July 2013) were designed by tiling (at 12×) along each genome and subsequently filtering to ensure even representation of distinct bait sequences across any given genome. Both actions were performed using in-house PERL scripts developed by the PATHSEEK consortium. The specificity of the baits was verified by BLASTn searches against the Human Genomic + Transcript database. The custom designed HCMV bait library was uploaded to SureDesign and synthesized by Agilent Technologies (San Diego, USA). Following library preparation and enrichment for HCMV DNA by hybridization using the SureSelect XT v1.5 protocol (200 ng starting material), modified only to increase the post-hybridization PCR yield where viral loads were low, deep sequencing using an Illumina MiSeq (500 bp v2 kits) was performed yielding millions of 2×250 bp paired-end reads. Paired-end reads were trimmed and mapped by local assembly against a database of all full or partially sequenced HCMV genomes available in GenBank. Several isolates of HCMV have been previously sequenced for their whole genome and the presence of hypervariable regions within the HCMV genome are well documented (Lurain et al. 1999; Rasmussen et al. 2002; Garrigue et al. 2007; Bradley et al. 2009), which led us to use a *de novo* approach to read assembly (Cunningham et al. 2010). Thus, all HCMV mapping read pairs were extracted and assembled into two *de novo* contigs forming the U_L_ and U_S_ sequences. Accurate assembly of the repeated regions (TR_L_, IR_L_, IR_S_, and TR_S_) could not be performed with short read data while insufficient material was available to obtain this sequence by other methods. A positive correlation was observed between the input viral load and the percentage of HCMV mapping reads (On Target Reads percent (OTR percent)), which was maintained until saturation i.e. the point at which the number of unique RNA baits is less than the total number of HCMV genome copies present in the hybridization reactions.

### 2.2. Consensus sequence analyses

Consensus sequences comprising the U_L_ and U_S_ regions for each sample were generated using a minimum read depth of 35 reads per base with low coverage regions coded as ambiguities (Ns). All consensus sequences were aligned against all available low/un-passaged (≤ 3 passages) HCMV genome sequences in GenBank using the program Mafft, v7 (Katoh and Standley 2013). The alignment was subsequently inspected by hand to correct sequence alignments in the hypervariable regions. Nucleotide diversity estimates were obtained with in-house R scripts using the 'ape' package (Paradis et al. 2004; Popescu et al. 2012). Phylogenetic network analysis were performed using SplitsTree4 (Huson and Bryant 2006).

### 2.3. Gene phylogenies

Newly sequenced genomes were annotated with RATT (Rapid Annotation Transfer Tool, version 18) (Otto et al. 2011) in reference to the annotation of reference strain Merlin (GenBank AY446894.2) with a 'Species' transfer setting, and were cross-checked with an analogous annotation using strain AD169 (GenBank FJ527563) as a reference, allowing the recovery of genes missing from the Merlin reference sequence, notably UL128. Coding sequence (CDS) alignments were obtained by first aligning the encoded protein sequences with ClustalOmega (Sievers et al. 2011) (version 1.2.1, default parameters) and then reverse-translated to CDSs with pal2nal (Suyama et al. 2006) (version 14), using Python scripts though the interface provided by BioPython modules (version 1.63) (Cock et al. 2009) and finally hand-corrected for incongruent alignments in repetitive regions using the SEAVIEW program (Gouy et al. 2010). Alignments of genes *RL12*, *UL50*, *UL146*, and *IRS1* were discarded because most sites in gene sequences were not homologous. Alignments were subsequently scanned for within-gene recombination breakpoints using the GARD algorithm from the HyPhy package (Kosakovsky Pond et al. 2006a, b), and the alignment was split according to significant breakpoints. Contrary to LD analysis, GARD screen does not have a site-scale resolution since it relies on regional signals to detect significant disagreement of the phylogenetic signals found on each side of the breakpoints (phylogeny reconstructed under the HKY85 substitution model, rejection of common history based on a Kishino–Hasegawa test, *P* < 0.05) (Kosakovsky Pond et al. 2006). However, it permitted us to ascertain that, in each (part of a) gene, any phylogenetic signal that is found with strong support is stemming from a unique vertical history. The 202 alignments obtained for each gene or gene part resulting from GARD analysis (hereafter simply called genes) were used for a Bayesian phylogenetic analysis using MrBayes (version 3.2.2) (Ronquist and Huelsenbeck 2003) under a GTR + I + 4Γ nucleotide substitution model and default parameters (notably two independent runs of four chains over 1,000,000 generations, including 250,000 discarded as burn-in and then sampling of trees every 500 generations).

We took advantage of the recent study by Sijmons et al. (2015) and the resource of gene alignments they provided to extend our dataset by merging Sijmons et al.'s gene alignments with those produced in the present study (split at the same recombination breakpoints as determined previously), using MAFFT--merge option (Katoh and Standley 2013). This resulted in a set of 181 alignments with at most 142 sequences (some genes being missing in certain strains) which we used to replicate all downstream analysis, exception made of the BranchSiteREL positive selection scan (see below). In that case, the smaller dataset is sufficiently representative of the species' diversity to cover all major deep splits, whereas the larger number of branches per tree in the extended dataset (ca. 300) would have unnecessarily lowered the power of the branch-wise test. For both datasets, lists of covered loci, their alignments and gene trees are available on TreeBASE server at: http://purl.org/phylo/treebase/phylows/study/TB2:S18391.

To compare phylogenetic histories of different gene loci, the diversity of phylogenetic signals recorded in HCMV genomes was explored by building a matrix of posterior probability (PP) supports of tree bipartitions observed in each gene's MCMC sample of trees (Supplementary Fig. S1). Linkage between neighboring genes was identified as tracks of genes with shared high PP for the same bipartition(s). This method enables the recognition of similar clusters of strains along the genome, signing their recent history of common descent. In particular this approach does not require that all sequences share the history of descent across loci (i.e. that gene tree topologies are completely matching) and hence allows identification of haplotypes shared by a group of strains where other strains are recombining. A similar matrix can be obtained for the compatibility score of bipartitions with the gene alignment, defined as one minus the maximal PP support observed for any bipartition that is topologically exclusive (incompatible) with the bipartition of interest (Supplementary Fig. S1). The latter approach allows a more sensitive scan for shared histories among genes as it detects non-exclusive signals from non-fully resolved parts of trees, and, at the same time, it can reveal gene alignments in which no signal is contained as they will not reject many or all possible bipartitions (vertical blue smears in Supplementary Fig. S1).

#### 2.3.1. Recombination and LD analyses

To perform an LD scan at the genomic scale, a 238,390-bp alignment of the 42 genomes dataset (excluding the TR_L_, IR_L_, IR_S_, and TR_S_ regions) was restricted to its 22,159 bi-allelic positions having at most one missing sequence. LD scans were performed using in-house R scripts making use of the 'ape' package (Paradis et al. 2004; Popescu et al. 2012). Significance of LD was tested using Fisher's exact test on the 2×2 contingency table of counts of possible allele pairs. First, we searched the all-versus-all biallelic site matrix for significant associations at *P* < 0.05, using the Bonferroni correction for multiple testing. Then, to detect short domains resilient to recombination, a sliding window scan was conducted to detect local peaks of LD (windows of 20 bi-allelic sites with a sliding step of 5 sites). The −log10 transform of Fisher's test *P* values [−log10(*P*_LDFisher_)] from a window were compared to the distribution of −log10(*P*_LDFisher_) for the genome-wide set of comparisons made in this range (i.e. within the diagonal ribbon of the square matrix of all-versus-all sites LD tests, sampled with a quantile function to get a representative sample of the same size than the window's) using a Mann–Whitney–Wilcoxon U test (single-tailed test with alternative hypothesis of local LD greater than the background). The reported local LD index is defined as −log 10(*P*_Wilcox_), with *P*_Wilcox_ the *P* value of the Mann–Whitney–Wilcoxon U test. Using windows of 20 successive bi-allelic sites spreading over variable physical distances (window sizes ranged from 24 bp to 3,376 bp), we observed that the density of bi-allelic SNPs was strongly correlated to the power of the test (Pearson's correlation, *r *=* *0.56, *P* < 10^−^^16^). Because the physical spacing of sites impacts directly the expected linkage between them, we modified the scan so that bi-allelic SNP density is constant and cannot bias our measure of LD: we used windows of fixed physical size in which a constant number of 20 bi-allelic sites were sub-sampled so that their spacing is the closest possible to uniformity. We chose a window size of 700 bp as a trade-off between resolution and coverage of the genome (95 percent of windows encompassed at least 20 sites) (Supplementary Fig. S2).

#### 2.3.2. Simulation studies

To validate the properties of the local LD index, and notably to investigate its dependence on nucleotidic diversity, we performed simulations of genome evolution using the software ALF (Dalquen et al. 2012). We generated synthetic genomes where nucleotidic diversity is homogeneous, but population structure and recombination rate varies among loci. On one hand, 21 background loci of ∼500 bp were simulated following trees that have no strong clonal structure and underwent homologous recombination during evolution, with different histories of orthologous replacement events in each of their five ∼100-bp partitions; this yielded a background of mosaic phylogenetic history marked by recombination (expected to induce strong homoplasy signatures). On the other hand, three interspersed foreground loci of size ∼500 bp were simulated following trees that show strong clonal structure (i.e. tight clusters of strains at the tip of long internal branches, expected to induce strong LD patterns) and did not undergo recombination during evolution. The resulting simulated genomes of size ∼12,000 bp thus meet the conditions for local LD test to be applied – searching for relatively rare local hotspots of high LD in a background of little or no LD – without the confounding effect of covarying nucleotidic diversity. To ensure that the simulation conditions were the closest to the real conditions of our HCMV dataset, gene trees from the 42-genome HCMV dataset were used as guides to the simulation, yielding alignments reflecting population structure that vary in a way comparable to the HCMV dataset. All loci evolved under the same evolution model (CodonPAM) with trees of similar sizes, producing a homogeneously distributed range of nucleotidic diversity values (ranging 0.07–0.23), allowing us to see the potential covariation of factors under a range of parameter combinations (low/strong diversity with low/strong population structure). Parameter files and guides trees are available in Supplementary File S1.

#### 2.3.3. Population structure

The 42-strain genome-wide alignment of bi-alleic SNPs described above was used to perform a PCA on the covariance matrix of bi-allelic genotypes. The latter analyses was repeated after further restricting the dataset to the 5,669 bi-allelic positions having at most one missing sequence and a minimum of 3 counts for the minor allele, to focus on the variation of ancient polymorphisms. To remove the effect of the strong linkage in the RL10–UL13 region in structuring the signal from the whole genome, we recomputed both phylogenetic networks and PCA separately for the whole-genome alignment restricted to the RL10–UL13 region (positions 8,600–21,100 in Merlin coordinates, 2,347 bi-allelic sites and 812 bi-allelic sites with a minimum of 3 counts for the minor allele, respectively) and for the remainder of the genome. The R code used for these analyses is available at https://github.com/flass/genomescans/.

### 2.4. Genotype classification

To characterize the extent of intergenic linkage when considering all strains together, we categorized strains into genotypes for each gene locus, using an iterative approach based on the variance of branch lengths for recognizing the relatively long branches in a gene tree. Clades in midpoint-rooted Bayesian gene trees were systematically evaluated from the smallest (skipping those made of less than five tips) to the most inclusive ones: the variance *Vc* of all branch lengths in the clade's subtree were computed, and then compared to the variance *Vp* computed when adding the parent branch; if *V*p > 2 · *V*c and the parent's branch length was greater than the mean branch length in the subtree and the parent branch had a minimum PP support of 0.8, the parent branch was pruned from the gene tree. Similarly, each branch within the clade was systematically singled out to test if it increased the variance of the subtree's branch lengths, and if so was pruned accordingly. This step was repeated after pruning until no evaluated branch was to prune in the evaluated clade, then higher clades were explored, until reaching the root of the gene tree. This procedure yielded for each gene tree a forest of subtrees, which defined clusters of strains, or genotypes. To avoid later statistical biases when comparing gene trees with heterogeneous numbers of genotypes, the number of genotypes to obtain was fixed to 8 (median of unrestricted genotype counts over all genes), meaning that the iterative pruning procedure was stopped when reaching this number. Alternatively, if not enough significant genotypes were found on the first pass, the procedure was repeated on all branches, including new branches resulting from the previous pruning steps, with a sensitivity increased by halving the thresholds of the test described above. The final set of genotypes is indicated in Supplementary Table S1. This approach and the parameters used here were designed to produce clusters of significantly diverged strains independently of the variable evolutionary rate of each gene. Profiles of genotypes were then compared between all gene loci using the RV matrix correlation statistics (Robert and Escoufier 1976; Josse et al. 2008). Correlation *P* values were scaled using the Bonferroni correction, accounting for the *N ·* (*N* – 1)/2 multiple tests. All phylogenetic tree manipulations were implemented using the 'tree2' Python package (github.com/flass/tree2/) and all statistical analyses were performed with R statistical software, with notable use of 'ade4' and 'ape' packages (Dufour and Dray 2007; Popescu et al. 2012).

#### 2.4.1. Positive selection

Positive selection scans were performed for each gene locus using the adaptive BranchSiteREL (aBSREL) algorithm provided in the HyPhy package (Kosakovsky Pond et al. 2011; Smith et al. 2015). This program allows searching for branches in a gene tree where the *dN*/*dS* ratio is greater than one for a significant proportion of the sites in the alignment, without *a priori* on the branches to test, thus highlighting every episode of positive selection that may have affected any set of sites in the gene sequence. aBSREL was run on the topology of the Bayesian gene tree; the alpha parameter was fixed per branch and 0.05 was set as threshold of corrected *P* value for significance. An algorithm of the same family but with greater statistical power, BUSTED, was recently published as a more appropriate tool to detect the occurrence of any episode of positive selection in at least one of the positions of the alignment (Murrell et al. 2015). We, therefore, applied this test to the same dataset and to the extended (142 strains) dataset. Both tests yielded comparable results, as 29 genes were found to carry signals of past positive selection episodes using BUSTED out of the 33 that had been detected by aBSREL (Supplementary Table S2). We conservatively considered a gene under positive selection when positive for both the aBSREL test and the BUSTED test on at least one of the datasets (Supplementary Table S3). aBSREL result reports were then analyzed to identify which branch in the gene tree were identified as episodes of positive selection (Supplementary Table S2) and to compute the branch-weighted average *dN*/*dS* per gene (Supplementary Fig. S2).

#### 2.4.2. Inter-specific genome comparisons

Translated proteomes from representative genomes from six other species of primate-infecting cytomegaloviruses (CCMV – Chimpanzee CMV, NC_003521; RhCMV – Rhesus macaque CMV, NC_006150; GMCMV – Green Monkey CMV, NC_012783; CyCMV – Cynomolgus macaque CMV, NC_016154; OMCMV – Owl Monkey CMV, NC_016447; SMCMV – Squirrel Monkey CMV, NC_01644), as well as MCMV (Murine CMV, NC_004065) were searched for homology to HCMV (NC_006273) proteome using BLASTP. A multiple genome alignment of the eight genomes was obtained using progressiveMauve (version 2.3.1) (Darling et al. 2010) under the default parameters plus the collinear flag, and the core parts of the alignment were used to build a species phylogeny with RAxML (version 7.7.2) under a GTR + I + 4Γ nucleotide substitution model (Stamatakis 2006).

## 3. Results

### 3.1. Origin and characteristics of the genomic dataset

To enable analyses of recombination amongst circulating HCMV genomes, 26 whole genome sequences were retrieved from GenBank, comprising 17 low-passage and nine uncultured strains (Bradley et al. 2009; Cunningham et al. 2010; Dargan et al. 2010; Jung et al. 2011; Sijmons et al. 2014). To increase the heterogeneity of the dataset relative to the worldwide HCMV diversity, 16 new sequences (nine uncultured and seven single-passage clinical samples) were generated through the PATHSEEK pipeline (http://www.ucl.ac.uk/pathseek), which uses a target enrichment method to enable deep sequencing of HCMV DNA directly from the clinical material (Depledge et al. 2011, 2014). The samples collected were primarily from congenitally infected (immune-competent) neonates and immune-deficient adolescents/adults (full sample details are shown in the 16 first rows of Table 1). While the complete dataset is dominated by HCMV strains isolated from Western Europe, individual sequences from the USA and South Korea were also included.

The repeat regions flanking the unique long (U_L_) and unique short (U_S_) were excluded from all downstream analyses, as accurate sequences could not be generated from next-generation sequencing data alone for the 16 additional samples. Nucleotide diversity between consensus sequences of individual strains were in the interval 0.46–3.26 percent (average, 2.49 percent) for the whole dataset and no significant difference was observed between low-passage strains (0.46–3.26 percent, average 2.49 percent) and uncultured samples (1.40–3.04 percent, average 2.45 percent, *t*-test, *df *=* *427, *P* > 0.17). By remapping the read data for each sample against its consensus sequence, estimates of nucleotide diversity for within-host populations (i.e. the diversity within a single patient sample) could be derived. Prior studies initially reported the nucleotide diversity of within-host HCMV populations to be 0.210–0.640 percent, comparable with that of the +ssRNA Dengue virus (Renzette et al. 2011). While this estimate was subsequently reduced to 0.080–0.200 percent (Renzette et al. 2013), here our estimates are lower again (0.016–0.059 percent, mean 0.0306 percent), with the greatest diversity observed in samples obtained from whole blood (Supplementary Fig. S4).

### 3.2. Variety of phylogenetic signals demonstrates homologous recombination extensively shapes HCMV genomes

Given the diversity of origins and clinical characteristics of samples in our dataset, we investigated whether there were genetic properties shared by subsets of our HCMV genome sample that might indicate epidemiological structure and/or adaptation of sub-populations to specific niches. A multiple sequence alignment comprising the 42 HCMV genome sequences was generated to study the genome-wide population structure. Principal component analysis (PCA) of polymorphism at bi-allelic sites did not show any obvious population structure and samples did not cluster by any of the available epidemiological factors like geographic origin, body compartment and host immune status (Supplementary Fig. S5A and B and Supplementary File S2). These findings were confirmed by the reconstruction of a phylogenetic network based on the whole-genome alignment, where no clade showed a clear separation from the rest of the population (Supplementary Fig. S6A). The absence of structure appears to be due to extensive homologous recombination, as evidenced by the numerous central reticulations of the network involving virtually all combinations of lineages as recombination partners. The role of recombination in mixing the genetic pool of HCMV at the whole genome level is confirmed by a highly significant rejection of the pairwise homoplasy index (PHI) test (Bruen et al. 2006; *P* < 10^−^^8^).

To understand how recombination shapes the diversity of HCMV genomes across loci, we reconstructed the phylogenetic history of each gene locus in the HCMV genome and looked for consistency of strain relationships across the genome. These phylogenetic inferences require prior definition of non-recombining loci in the genome. To identify these regions, we used GARD (Kosakovsky Pond et al. 2006) to screen the alignment of each annotated gene for the presence of segments of sequence showing incongruent phylogenetic signals due to recombination; in the presence of recombination breakpoints, the gene alignment was split into sub-alignments carrying statistically uniform phylogenetic signal. The resulting collection of alignments was used to build Bayesian phylogenetic trees. The phylogenetic signal at each locus was then dissected into every tree bipartition recorded in the sample of gene trees, to quantify the support for any cluster of strains (Supplementary File S3). By comparing their occurrence between neighbouring loci, we could reveal haplotypes shared by groups of strains of various sizes. The majority of loci showed no consistent phylogenetic patterns with their neighbors, proving that recombination was recurrent through HCMV history, yielding conflicting patterns of strain relationships between loci. The signature of the most recent shared ancestry of strains (by either clonal descent or recombination), i.e. the pairing of strains as closest relatives in the gene trees, formed haplotypes no longer than two genes on average (Supplementary Fig. S7). This shows that recombination occurs frequently enough to yield genomes that behave as gene-scale mosaics of sequences of different origin.

Nonetheless, rare haplotypes spanning long stretches of the genome were shared by pairs of strains, marking recent recombination events (Supplementary Fig. S8). In addition, several long haplotypes involving larger groups of strains could be recovered: one 22 gene-long haplotype ranging from gene *US1* to gene *US24*, shared by strains 3,157, BE/10/2010 and NL/Rot1/Urine/2012 (Supplementary Fig. S1A, black arrow), and a few haplotypes at the left hand end of the prototypic genome orientation (genes *RL10* to *UL13*; Supplementary Fig. S1A and B, top left of each panel), indicating that some level of population structure occurs locally in the genome. This was confirmed by repeating the PCA and phylogenetic network analysis on the segment of the whole-genome alignment covering the RL10-UL13 genomic region, revealing three major clusters of diversity in this region (Supplementary Figs. S5C and D and S3B), while diversity in the remainder of the genome appear even less structured when considered separately (Supplementary Figs. S5E and F and S3C).

We reran the gene-scale phylogenetic analysis after inclusion of the dataset of gene alignments recently published by Sijmons et al. (2015) which leads to a total of 142 strains. The inclusion of these additional sequences allowed for a better resolution of gene tree topologies, with an average ratio of bipartitions posterior probability support over compatibility of 0.056 in the 42-genome dataset vs. 0.169 in the 142-genome dataset (Supplementary Figs. S1D vs. S1B), thus increasing the method's specificity when identifying common haplotype structures across loci. In this more conservative setting, we could nonetheless detect many long haplotypes up to the size of the genome shared by several sets of strains, e.g. by BE/1/2011, BE/8/2010, and BE/9/2012. This indicates clonal relatedness of these strains, as could be expected from the clustering in time and space of the samples from Sijmons et al. (2015).

### 3.3. Linkage disequilibrium shows heterogeneous intensity of recombination at genome-wide and sub-genic scales

While it is beyond doubt that HCMV genomes undergo frequent and extensive recombination, we next asked whether this process is equally frequent throughout the genome. We thus used linkage disequilibrium (LD) approaches to study the intensity of recombination between loci. LD reflects the non-random association of alleles at two loci, with a high LD value indicating co-inheritance of loci (Slatkin 2008) and hence lower incidence of recombination between them. LD is usually characterized based on the distribution of biallelic single nucleotide polymorphisms (SNPs), but can also be inferred using a categorization of DNA sequences into alleles or haplotypes. We thus conducted two sets of LD scans to evaluate the impact of recombination on population structure at different scales in the HCMV genomes.

**Figure 1.**
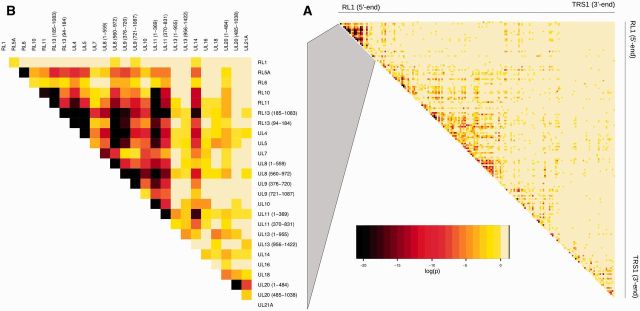
Heat map of correlation significance between the distribution of sequence genotypes in the 142 strains along (A) the whole genome or (B) in a close-up of the first 19 genes (25 non-recombining loci) in the prototypic HCMV genome organization. *P* values are indicated by coloring of the matrix cells (see Supplementary methods for attribution of genotypes). Each individual gene alignment was first scanned using GARD to identify recombination breakpoints and the alignments subsequently split either side of the breakpoint and considered as separate entities.

**Figure 2.**
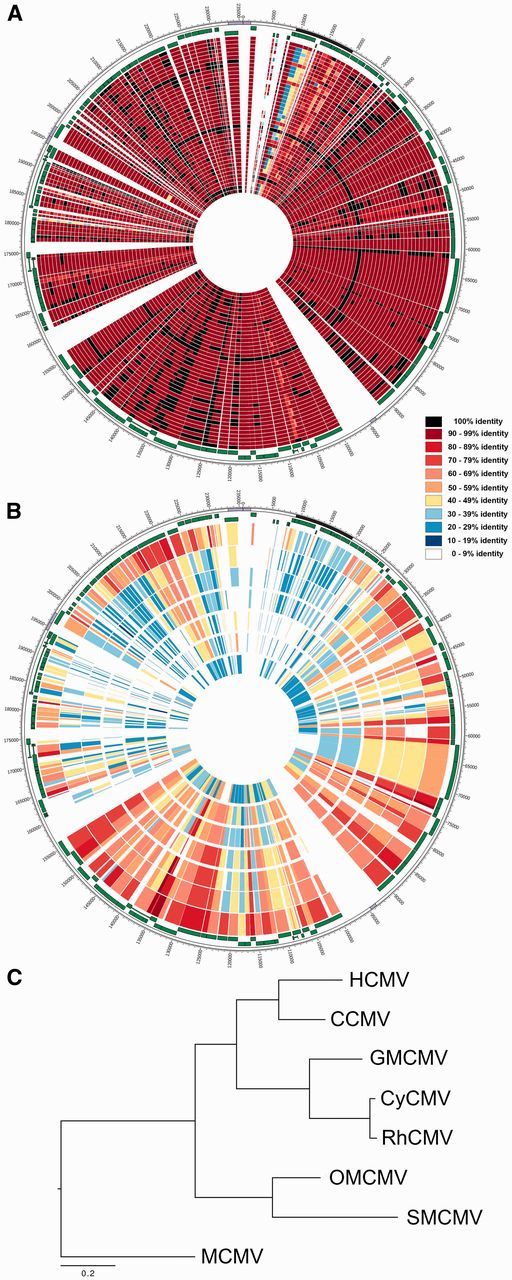
Conservation of protein sequences in cytomegalovirus. Syntenic circular genome maps showing protein sequence conservation (percent identity) against HCMV strain Merlin for (A) all 42 HCMV strains used in this study and (B) the non-human CMVs, from outer to inner track: CCMV, GMCMV, CyCMV, RhCMV, OMCMV, SMCMV, and MCMV. The percentage sequence identity is illustrated by the color legend for both A and B. (C) Maximum-likelihood tree of eight cytomegalovirus species (as in (B)) based on a whole genome alignment of conserved syntenic blocks. All bootstrap support values (based on 100 samples) were 100 percent.

First we sought potential linkage between genes across the genome. Gene sequences were classified into genotypes using an algorithm for dynamic pruning of gene trees from the 142-genome dataset (see Material and methods section), and these categories were used to identify genome-wide correlation of genotype distribution among strains, yielding a metric analogous to LD. The majority of genes showed no significant association with genes other than their immediate neighbor (Fig. 1A). The most striking exception to his trend is the cluster of 13 genes (*RL10* to *UL11*, included in the structured RL10–UL13 region reported above) in substantially higher LD, consistent with locally reduced recombination (Fig. 1B). The average linkage between direct neighbour genes in this region is *r*^2 ^=^ ^0.45, and the most distal genes in the locus, RL10 and UL11, are 9 kb apart and yet are in tighter linkage (*r*^2 ^=^ ^0.34) than the average for pairs of direct neighbor genes elsewhere in the genome (*r*^2 ^=^ ^0.18). This region includes multiple genes from the RL11 family and is hereafter referred to as the RL11 region. A comparative similarity analysis of the HCMV proteomes demonstrates substantial variation in this region (across the protein-coding loci in the region, the most divergent pairwise strain comparisons range from 30 percent to 70 percent protein identity) (Fig. 2A). Moreover, when extending this comparison to include all primate CMV and murine CMV species, this region consistently retains the highest genetic variability (Fig. 2B). A second gene cluster of 11 genes (*UL87*–*UL97*) also shows a significant degree of LD (Fig. 1C), although to a lesser extent (average linkage between direct neighbours, *r*^2 ^=^ ^0.23). To verify that these results do not only stem from the clonal relationships between a few related strains in the dataset, we split the dataset into two subsets, the first comprising the 42 worldwide genomes and the second the 100 genomes from Sijmons et al. (2015), which were collected on a more restricted geographic and temporal scale (Supplementary Fig. S9). In both cases, we robustly recovered the high linkage of RL11 region. However, the linkage within the *UL87*–*UL97* cluster is only evident within the larger dataset from Sijmons et al., in which it may reflect the clonality of a few strain clusters. This hypothesis is supported by a group of 13 strains sharing a large haplotype that covers this region, among which twelve were sampled in Belgium and ten in the same year (Supplementary File S3, bipartition #2484). 

To characterize the recombination landscape at a finer scale, we performed a genome-wide LD scan, based on the polymorphism at bi-allelic sites, i.e. sites where exactly two different nucleotides are present in the sample. In order to characterize the genomic properties common to the whole viral species and to exclude the potential effect of clonal population structure, we restricted our analyses to the dataset of 42 diverse genomes. A total of 22,159 bi-allelic sites were identified within the 238-kb whole-genome alignment. Using Fisher's exact tests for all pairwise site comparisons, we identified 19,084 significantly linked site pairs (Bonferroni-corrected *P* values < 0.05). The maximal distance between linked sites was 16.35 kb, with 99 percent of the distances falling below 3 kb and 87 percent below 1 kb. The latter analysis points to the absence of long-range linkage among most sites of HCMV genomes and supports widespread intra- and inter-genic recombination.

**Figure 3.**
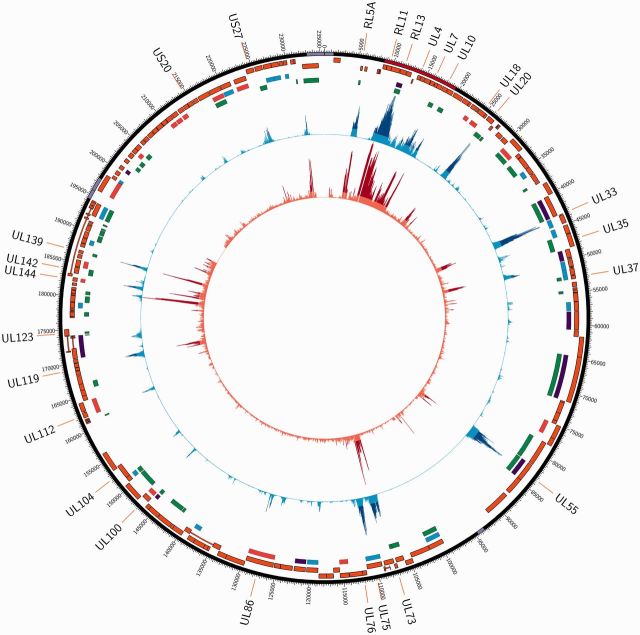
Circular genome map showing linkage disequilibrium and nucleotidic diversity. (A) The purple backbone represents the classical HCMV genome arrangement TR_L_–U_L_–IR_L_–IR_S_–U_S_–TR_S_ where repeat sequences are shown in a lighter shade, the origin of lytic replication highlighted in pink, and the RL11 gene family indicated by the red shading. Tracks are numbered inwards: (1) Map of the protein-coding genes; (2) Presence of epitopes for CD4+ (blue), CD8+ (red) or both (purple)T-cells (Sylwester et al. 2005); (3) location of genes that have undergone positive selection episodes; (4) Local LD index, computed in 700-bp windows, the hotspots of LD (top 5 percent values) are highlighted in dark blue, with corresponding gene names shown outside of the plot; (5) HCMV nucleotide diversity, computed in adjacent windows of 100 bp, the hypervariable loci (top 5 percent values) are highlighted in dark red.

Given the near-absence of long-range linkage in the HCMV genomes, we next searched for short-scale patterns of excessive LD using a local LD index (see Methods section) in sliding windows of 700 bp over the whole genome alignment (Supplementary File S4). We identified hotspots of linkage in 31 genes out of the 169 annotated in the Merlin reference genome, as those harboring the top 5 percent windows in higher LD than the genomic average (corresponding to local LD index > 5) (Fig. 3A, blue track 4). Interestingly, there is an apparent association between these hotspots of LD and peaks of nucleotide diversity, a measure of average similarity between strains in our sample (Fig. 3A, red track 5; Supplementary Table S4). Indeed, out of the 26 hyper-variable genes (those harbouring the top 5 percent windows of higher nucleotide diversity), 21 were also found to be LD hotspots (Table 2). This association is confirmed by a significant correlation between nucleotide diversity and the *r*^2^ metric of LD (Pearson's correlation, *r*^2 ^=^ ^0.214, *P* < 10^−^^16^) or the local LD index (*r*^2 ^=^ ^0.307, *P* < 10^−^^16^). 

**Table 2.** **** List of genes with the strongest hotspots of linkage disequilibrium (high-LD genes)

Gene^a^		Functional Annotation^b^	CD4+^c^	CD8+^c^	LD score^d^	Nuc. Div.^e^
*UL33*		Envelope glycoprotein, modulation of chemo- and/or cytokine receptor through binding (CCR5/CXCR4)		+	**32.9**	**0.16**
*RL13*	#	Glycoprotein, repression of replication	++	+	**32.2**	**0.41**
*UL20*		Membrane glycoprotein, modulation of T-cell signalling/function			**28.9**	**0.19**
*UL55*		Glycoprotein B (gB), heparan-binding, viral entry of the host cell	++	++	**26.6**	**0.16**
*UL75*		Glycoprotein H (gH), viral entry of the host cell	+	+	**24.4**	0.09
*RL6*	#	Putative membrane glycoprotein			**20.1**	**0.38**
*UL139*	*	Putative membrane glycoprotein sharing sequence homology with CD24			**18.1**	**0.38**
*UL9*	#	Putative membrane glycoprotein			**17.9**	**0.32**
*UL8*	#	Putative membrane glycoprotein			**16.5**	**0.20**
*UL73*		Glycoprotein N (gN)			**14.4**	**0.30**
*UL11*		Membrane glycoprotein, modulation of T-cell signalling/function			**14.0**	**0.34**
*UL120*		Putative membrane glycoprotein			**12.3**	**0.22**
*UL86*		Major capsid protein (forms icosahedral capsid with UL85, UL80, UL48/49, and UL46)	++	+	**12.1**	0.02
*UL7*	#	Membrane glycoprotein, modulates chemo- and/or cytokine production			**10.9**	**0.12**
*UL37*		Membrane glycoprotein, MHC-I homologue, mitochondrial inhibitor of apoptosis (vMIA)		++	**10.6**	**0.18**
*US27*		Membrane glycoprotein, predicted involvement in virion assembly and egress			9.8	**0.14**
*RL5A*	#	Putative membrane glycoprotein			9.8	**0.23**
*UL10*		Putative membrane glycoprotein			9.8	**0.12**
*UL144*	*	Type I transmembrane glycoprotein and a potent activator of NFκB-induced transcription	+		9.3	**0.27**
*UL112*		Transcriptional activator involved in DNA replication			8.6	0.04
*UL100*		Glycoprotein M (gM), viral entry of the host cell	++		8.5	0.05
*UL35*		Tegument phosphoprotein			8.0	0.03
*UL4*	#	Putative membrane glycoprotein			7.4	**0.16**
*UL119*		membrane glycoprotein, binds IgG Fc domain, involved in immune regulation			7.4	**0.12**
*RL11*	#	membrane glycoprotein, binds IgG Fc domain, involved in immune regulation			6.7	**0.20**
*UL123*		Transcriptional regulator IE1, involved in immune regulation	++	++	6.7	**0.05**
*UL142*	*	Putative membrane glycoprotein (homology to MHC-I) with NK cell evasion function			5.5	**0.14**
*UL104*		Capsid portal protein			5.5	0.02
*UL76*		Virion-associated regulatory protein			5.0	0.06
*UL18*		Putative membrane glycoprotein (homology to MHC-I) with NK cell evasion function	++		5.0	0.06
*US20*		Putative multiple transmembrane protein			5.0	0.03

#, RL11 family; *, UL/b’, region.

^a^Gene name in Merlin reference sequence annotation (NCBI RefSeq NC_006273/AY446894), genes are ranked by decreasing LD score.

^b^Functional annotation summarized from Merlin reference sequence annotation in NCBI RefSeq record (NC_006273/AY446894) and from the extensive review provided by van Damme and van Looke (2014).

^c^Antigenic status derived from Sylwester et al. (2005).   ++, the gene was among the top 30, when ranked by total memory-corrected response, over 33 seropositive subjects;  +, the gene was eliciting a positive response in at least 4 of the 33 tested seropositive subjects.

^d^The highest local LD index among all windows included in the gene boudaries; local LD index is the −log10 transform of the *P* values of Mann–Whitney–Wilcoxon tests for each 700-pb windows located in the gene, under the null hypothesis that there is no higher LD in the window than in average in the genome. Genes presented in this table include the 5 percent top-scoring windows (high-LD genes); bold values are in the top 2 percent.

^e^Nucleotidic diversity, highest value recorded in 100-bp windows within the gene; bold values are in the top 5 percent (hyper-variable genes).

To exclude the possibility that this correlation resulted from a systematic bias in our method leading to measure higher LD in more diverse regions, we performed simulations of genome evolution using ALF (Dalquen et al. 2012) under a model where phylogenetic structure and recombination rate vary independently of diversity (see Methods section). Analysis of these simulated genomes showed that the local LD index accurately reports local domains of clonal structure (accuracy statistics over 20 independent simulations, given a detection threshold of local LD index ≥ 5): per-window sensitivity SN_w_ = 0.63–0.85 (median 0.85) and specificity SP_w_ = 0.98–1.0 (median 1.0); average per-locus sensitivity SN_L_ = 1.0 and specificity SP_L_ = 0.95 (Supplementary Table S5). In addition, we verified that this metric is not positively misled by local peaks of nucleotidic diversity, with only a non-specific correlation to local nucleotidic diversity (Pearson correlation, *r* = −0.15–0.17, median 0.04, significant associations at *P* < 10^−^^2^: 2/20 negative and 2/20 positive). In comparison, other metrics used as proxy for homoplasy were not able to report as accurately the clonally evolving loci and were strongly impacted by the local diversity levels (Supplementary Fig. S10 and Table S5).

### 3.4. Hyper-variable loci are under diversifying selective pressure leading to divergence between haplotypes

Homologous recombination is likely to be restricted between highly differentiated genotypes due to the inability of homologous strands to anneal, favoring further divergence. Their primary divergence may, however, have been driven by positive selective pressure, i.e. selection for changes in the resultant protein. To test this hypothesis, we scanned all HCMV gene trees using the aBSREL algorithm (Kosakovsky Pond et al. 2011; Smith et al. 2015) and show that the genomes evolved mostly under purifying selection, with an average d*N*/d*S* of 0.18 over their complete evolutionary history (branch length-weighted average of mean d*N*/d*S* values for all branches pooled across all genes). This constraint on the diversification of encoded proteins is also seen within hyper-variable genes (average d*N*/d*S* of 0.22) but to a lesser extent than in other genes (average d*N*/d*S* of 0.17, *t*-test *P* value < 0.003) (Supplementary Fig. S3). However, this analysis also pointed to evidence for episodes of positive selection (branches with significantly non-null proportion of sites with d*N*/d*S* > 1) in 29 genes (Fig. 3, track 4 and Supplementary Table S2), nine of which have also been identified as hyper-variable (Supplementary Table S4 and Supplementary File S5) and hotspots of LD (Table 2). There is a significant enrichment of positively selected genes in hyper-variable non-recombining genes (Chi-squared test, d*f* = 1, *P* < 0.003). It should be noted that this test is conservative given the greater possibility of spurious inference of episodes of positive selection in recombining genes by the phylogenetic selection scan. Indeed, when using an alternative method based on pairwise sequence comparisons that does not make assumptions about the phylogenetic history of samples (Yang and Nielsen 2000), we generally fail to detect positive selection in recombining genes (data not shown). Closer examination of the nine positively selected hyper-variable genes identified episodes of positive selection in the deep branches separating the diverged genotypes as well as on terminal branches (Supplementary File S5), in contrast with purifying selection occurring in the rest of branches (Supplementary Table S2). This supports a scenario of recurrent episodes of positive selection throughout the evolution of these genes rapidly generating sequence diversity, driving these loci to gradually diverge into almost non-homologous genotypes.

## 4. Discussion

### 4.1. Recombination is widespread in HCMV genomes but linkage occurs locally in association with peaks of diversity

Using a series of phylogenetic and population genetics analyses, we characterized recombination in HCMV genomes at increasing resolution and show recombination to be so pervasive across most of the genome that virtually every variable site segregates independently. In this respect, this viral species can be considered to behave as a single freely recombining worldwide population. This observation is in line with the absence of any apparent geographical or epidemiological structure in the HCMV population at the genome-wide level (Supplementary Figs. S5 and S6), as has previously been reported for individual gene markers (Haberland et al. 1999; Pignatelli et al. 2004; Bradley et al. 2008). Recombination of sub-genomic fragments has previously been observed for Epstein–Barr virus and also genome-wide for herpes simplex virus 1&2, VZV, and murine CMV (Walling et al. 1994; Lingen et al. 1997; Norberg et al. 2007; Norberg et al. 2011; Norberg et al 2015; Smith et al. 2013), and thus appears to be a hallmark of many herpesviruses. Recombination is indeed known to be a crucial step of the replication process in HSV-1 (Wilkinson and Weller 2003) and has been postulated to occur between superinfecting as well as reactivating genomes in HCMV (Chou 1989; Haberland et al. 1999; Steininger et al. 2005; Garrigue et al. 2007; Steininger 2007; Faure-Della Corte et al. 2010). The topology of long haplotypes observed between pairs of strains is suggestive of recombination occurring through large replacement events, for example, by crossing-over or joining replicated genome segments (Wilkinson and Weller 2003) rather than by localized gene conversion events. The recent study from Sijmons et al. (2015) also reported the occurrence of intense, genome-wide homologous recombination in HCMV genomes and similarly stressed the heterogeneity of the recombination intensity across the genome. However, the map of the recombination landscape resulting from their analysis differs markedly from ours. Using recombination breakpoint detection methods, they showed a globally higher prevalence of recombination in genes with higher diversity (Sijmons et al. 2015). These breakpoint methods are appropriate to identify partitions of sequence alignments with different phylogenetic history (Kosakovsky Pond et al. 2006) in order to treat them separately in downstream analyses – as we did ourselves for our gene-scale analyses. However, recombination breakpoints are generically identified from the conflicting phylogenetic signals estimated over their flanking segments of sequence. Breakpoint detection methods thus can only report a finite number of recombination events per locus, which are biased towards the most obvious, i.e. recent events. This limited account of recombination history thus leads to a biased estimate of recombination intensities. In addition, methods like GARD (Kosakovsky Pond et al. 2006), which involve the reconstruction and comparison of phylogenetic trees, can be afflicted by high rates of false positives in the face of strong evolutionary rate variation across a gene sequence; a situation often met in genes with hyper-variable domains but highly conserved otherwise (e.g. in UL55/gB). Finally, these methods, like all polymorphism-based analyses, are highly biased towards the detection of more recombination breakpoints in more variable genomic regions. Conversely, LD can be quantified at every variable site and naturally integrates the effect of recombination over long evolutionary times. For all these reasons, we feel that the analysis of variation of recombination intensities within HCMV genomes is better addressed by the approach we deployed, which is based on LD and was specially designed to control for heterogeneity in polymorphism density.

In contrast to Sijmons et al.'s findings, we find the majority of hyper-variable loci to be in high linkage. Specifically, within these high-LD loci, recombination between divergent genotypes is prevented, although recombination is still expected to occur between strains sharing the same genotype. Whereas occurrences of LD have previously been identified between genes of the RL11 region (Sekulin et al. 2007), which harbors exceptionally large haplotypes (Supplementary Fig. S1), we here report several other loci where occurrence of LD is only evident at a scale smaller than a gene and when contrasted with the genome-wide background of essentially free recombination. Paradoxically, many of these hotspots of LD occur in genes that were associated with previous reports of absence of linkage between genes, as they are mostly hyper-variable and can be used as highly discriminant epidemiological markers (Haberland et al. 1999; Pignatelli et al. 2004; Bradley et al. 2008), but the presence of linkage within them has been overlooked so far.

### 4.2. Islands of linkage arise by fast evolution under diversifying selection or slow evolution under putative epistatic constraints

Within this genome-wide pattern of essentially free recombination and strong genome conservation, some 31 loci do not recombine freely (Fig. 3). Two-thirds of these genes have evolved multiple genotypes that are sufficiently divergent to prevent recombination between each other (Table 2). Considering the functional annotation of these genes, most (24/31) of the high-LD genes are annotated as membrane glycoproteins that are either known or predicted to be involved in virus entry or immune evasion (Table 2). In addition, 10/31 harbor T-cell epitopes (Fig. 3A, track 2; Table 2), as previously determined by a systematic experimental scan (Sylwester et al. 2005). Functions implying protein– protein interaction with host factors and/or having antigenic status are suggestive of co-evolutionary dynamics driving the diversification of these proteins. In nine of these genes, phylogenetic analysis revealed evidence of past and present positive selections, which likely accounts for this extreme divergence (Supplementary Tables S2 and S4). For these nine positively selected hyper-variable genes, which include antigenic viral proteins like UL55/gB, this pattern is highly suggestive of selective pressure for escape of the host immune system driving sequence diversification. Causes of diversification of other hyper-variable proteins are less obvious, and may involve more complex scenarios. For instance RL11 gene family members encode putative glycoproteins (both membrane and cytosolic, Gabaev et al. 2011) that can impact virus growth in a cell type-specific manner, manipulate leukocyte adhesion and cytokine production (via interaction with CD229) or bind to immunoglobulin-Fc receptors postulated to contribute to epithelial cell tropism *in vivo* (Lilley et al. 2001; Atalay et al. 2002; Engel et al. 2011). As these proteins bind host receptors that are themselves polymorphic, negative frequency-dependent selection for a tighter interaction with the variety of ligands available in the host population could be driving their diversification. Another known cause of the emergence of LD is the competition for resources such as cellular receptors among co-infecting strains, promoting a stably diverse population of pathogens (Watkins et al., 2014); this mechanism promoting population structure is, however, expected to lead to long-range linkage among loci, which is not observed here (apart for the RL11 locus, see below).

LD hotspots were also present within ten genes (including UL75/gH, UL86/MCP, UL100/gM, UL104, and UL123/IE1) that are more conserved among HCMV strains, with an average nucleotide diversity only marginally higher than the genomic average (Supplementary Fig. S11). For these, lack of recombination cannot be explained by between-genotype nucleotide divergence, and we speculate that in these genes linkage may preserve key protein functions that rely on crucial intra-genic epistasis. In the case of UL86 and UL104, both capsid proteins, it seems plausible that strong conformational constraints in these proteins – for which stability is essential for virion formation and transmissibility – may lead to such strong selection for co-evolution of interacting residues that linkage between them could be maintained even in face of the high recombination rate. In principle, such haplotypes maintained in LD by natural selection alone for sufficient periods of time might evolve into hypervariable regions through the independent accumulation of mutations.

The identification of a set of genes with function that drove (and is likely still driving) their constant divergence may be valuable for research into new therapeutic strategies. Glycoproteins gB (UL55) and gH (UL75), both of which are prime targets for neutralizing antibodies and vaccine development, are encoded by high-LD genes. This raises the possibility that other proteins in this group may also be suitable targets for the development of new drugs or vaccines, for example glycoprotein gM. It is interesting to note that none of these hotspots overlapped with more recent revisions of the HCMV coding content (Supplementary Fig. S12), and thus these inferences are unlikely to come from selection on cryptic functions of the loci.

### 4.3. RL11 region diversification is coherent and uncoupled from the rest of the genome

The most striking of the linked hotspots is the RL11 region (comprising genes *RL11*–*UL11*), where strong linkage is found between thirteen genes. Although the high degree of linkage in this region has previously been noted (Dolan et al. 2004; Sekulin et al. 2007), our whole genome analyses reveal this is the only region of the HCMV genome showing any evidence of a consistent evolutionary history (Fig. 1). These uniquely large haplotypes in the HCMV genome could reflect immune pressure for co-segregation of antigenic alleles at multiple loci into non-overlapping combinations of antigens to avoid cross-immunity, a phenomenon which has been shown to occur in some bacterial pathogens (Watkins et al. 2014). However, the fact that these co-segregating genes sit contiguously in a globally hyper-variable region prevent us from distinguishing whether recombination in this region is counter-selected or neutrally prevented because homologous recombination cannot be initiated due to the excessive divergence of sequences throughout this region.

Phylogenetic analysis of core genes of genomes of CMV isolated from eight host species, including all sequenced primate CMVs and using murine CMV as an outgroup (Fig. 2C), generated a topology congruent with the accepted evolutionary relationships of the primate host species (Perelman et al. 2011). This supports a history of repeated co-speciation of virus and hosts, during which the *RL11* gene family expanded and evolved rapidly in a continuous process of diversification since the early CMV ancestor (Fig. 2B). This rapid evolution of the RL11 region continued within HCMV species, with high levels of diversification within and between species (Fig. 2A).

Phylogenetic analysis within HCMV indicates that several members of the RL11 family (RL13, UL9, and UL11) originally diverged under positive selective pressures (Supplementary Table S3). This suggests that these genes have an important functional role in HCMV biology, even though a number of genes encoded in the RL11 region are non-essential for viral replication *in vitro* (Atalay et al. 2002). The common feature of this gene family is the RL11 core domain (RL11D), which shares homology with the adenovirus E3 family of proteins (Davison et al. 2003; Gabaev et al. 2011) which are important for evasion of T-cell responses and inhibition of cell death. This putative function, in addition to the characterized roles of individual proteins in cell tropism, would further implicate the RL11 region as a crucial region for HCMV replication *in vivo*, notably through specific interactions with human receptors. Therefore, we hypothesize that the *RL11* gene family diversification has been an important driver of adaptive co-evolution of HCMV genomes within their hosts. From this perspective, the presence of divergent genotypes at this locus having lost the ability to recombine with each other, while free recombination is still occurring in the rest of the genome, is indicative of population differentiation occurring within HCMV species. More genomic and epidemiological data are needed to link this cryptic differentiation to demographic or selective processes.

### 4.5. Recombination enables selection to operate with varying dynamics across HCMV genomes

Apart from the few hyper-variable loci, the majority of the HCMV genome harbors only limited genetic diversity (Fig. 3), translating into very homogeneous proteomes (Fig. 2A). This high degree of conservation at the protein level is likely due to the strong purifying selection experienced by all protein-coding genes in the genome over most of their phylogenetic history (Supplementary Fig. S3). Genome-wide purifying selection is common in viral pathogens (Wertheim and Kosakovsky Pond 2011; Wertheim et al. 2013; Cloete et al. 2014; Duchêne et al. 2014), indicating strong and long-standing evolutionary constraint on the encoded proteome. In HCMV, such constraint might be explained by the long co-evolution with its host: herpesviruses have co-evolved over long periods of time with their specific host (McGeoch et al. 1995; Wertheim et al. 2014) – in the case of CMVs with no evidence of further introductions since the last primate ancestor (Fig. 2C). With such extended time periods under constant ecological conditions, the HCMV genome is likely close to encode an optimal phenotype, characterized by a life cycle of asymptomatic reactivation and transmission. This lifestyle necessitates a large apparatus of regulators and immune escape factors that constitute as many mutation targets. Intense homologous recombination is known to increase the efficiency of natural selection by unlinking selected sites from the genomic background, and may thus provide the means to achieve the levels of purifying selection required for maintaining HCMV's genome functionality.

Moreover, we observe two sets of sites in HCMV genomes with conflicting evolutionary dynamics: those under strong pressure for long-term conservation, and those under episodic pressure for rapid diversification. In the presence of genetic linkage, selective pressures operating at independent loci interfere with each other and prevent fixation of the fittest genotypes, notably through the accumulation of deleterious mutations in the genomic backgrounds that carry positively selected mutations – the so-called Hill-Robertson effect, a counter-adaptive phenomenon avoided when recombination occurs between loci (Felsenstein 1974). This common evolutionary trade-off is emphasized in HCMV by the extreme dichotomy of evolutionary rates observed in the genome, with the few loci under strong positive selection for frequent change being at risk of recurrently hitch-hiking deleterious mutations in the large remainder of the genome under purifying selection. The maintenance of such contrasting evolutionary dynamics in a same genome prompts an even greater need for HCMV genomes to recombine frequently.

Population-level selection could thus have operated to favor intense genetic mixing. For instance, inter-strain homologous recombination is likely to occur during infection with multiple CMV strains, either simultaneously (co-infection) or over time (super-infection), phenomena which are frequently observed in both humans (HCMV) and mice (MCMV) (Booth et al. 1993; Meyer-König et al. 1998). Interestingly, evasion of pre-existing adaptive immunity, mainly CD8 T-cells, orchestrated by the *CMV US2-11* gene products, which down-regulate MHC class I and II antigen presentation pathways, has been shown to be critical for super-infection in the rhesus CMV primate model (Hansen et al. 2010). However, these genes are dispensable for primary infection of CMV-naive animals, and thus appear to specifically promote immune evasion during super-infection. Under the hypothesis of the crucial role of recombination in HCMV evolution, it would seem plausible that acquisitions of genes promoting super-infection and, as a consequence, frequent recombination of viral strains, were positively selected.

## 5. Conclusion

In summary, we conclude that HCMV recombines essentially freely, except at hotspots of LD found in 31 genes, among which a majority of hyper-variable genes that originally diverged under positive selection and code for proteins associated with adaptive or innate immune evasion. Immune evasion is postulated to favor the high levels of HCMV superinfection observed in humans, and this in turn promotes recombination. This mechanism would allow HCMV genomes to satisfy an evolutionary trade-off between short term pressures to evade host immune recognition and a long-standing evolutionary constraint on a complex genome optimally tuned towards silent parasitism.

## Data availability

New HCMV genome sequences were deposited in GenBank under the accessions KT726940-KT726955. Data used for and resulting from phylogenetic inferences are available on TreeBASE at: http://purl.org/phylo/treebase/phylows/study/TB2:S18391. Supplementary material is accessible on Figshare under Project no. 13369: Supplementary Tables S1–S5, DOI: 10.6084/m9.figshare.3219184; Supplementary Figures S1–S12, DOI: 10.6084/m9.figshare.3219214; Supplementary Files S1–S5, DOI: 10.6084/m9.figshare.3219223.

## Supplementary data

 Supplementary data are available here.

## Supplementary Material

Supplementary Data
