# Supplementary figures and images for: Islands of linkage in an ocean of pervasive recombination reveals two-speed evolution of human cytomegalovirus genomes
Source: Virus Evol. 2016 Jun 15;2(1):vew017. doi: 10.1093/ve/vew017 (PMC6167919; doi:10.1093/ve/vew017)

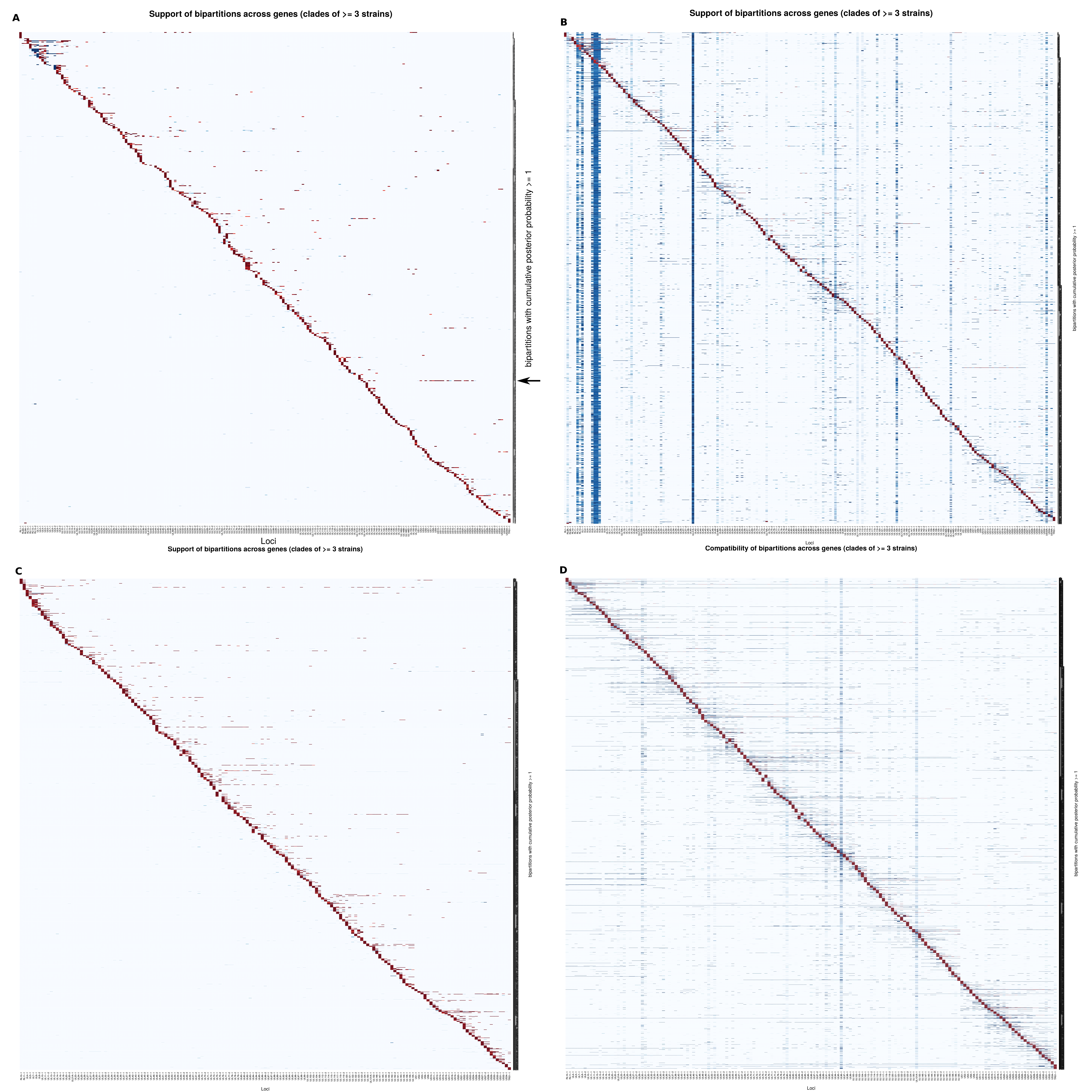

Supplement: Supplementary Data [file vew017_Supplementary_Data.zip › Sup_Figs/Figure S1.tif]

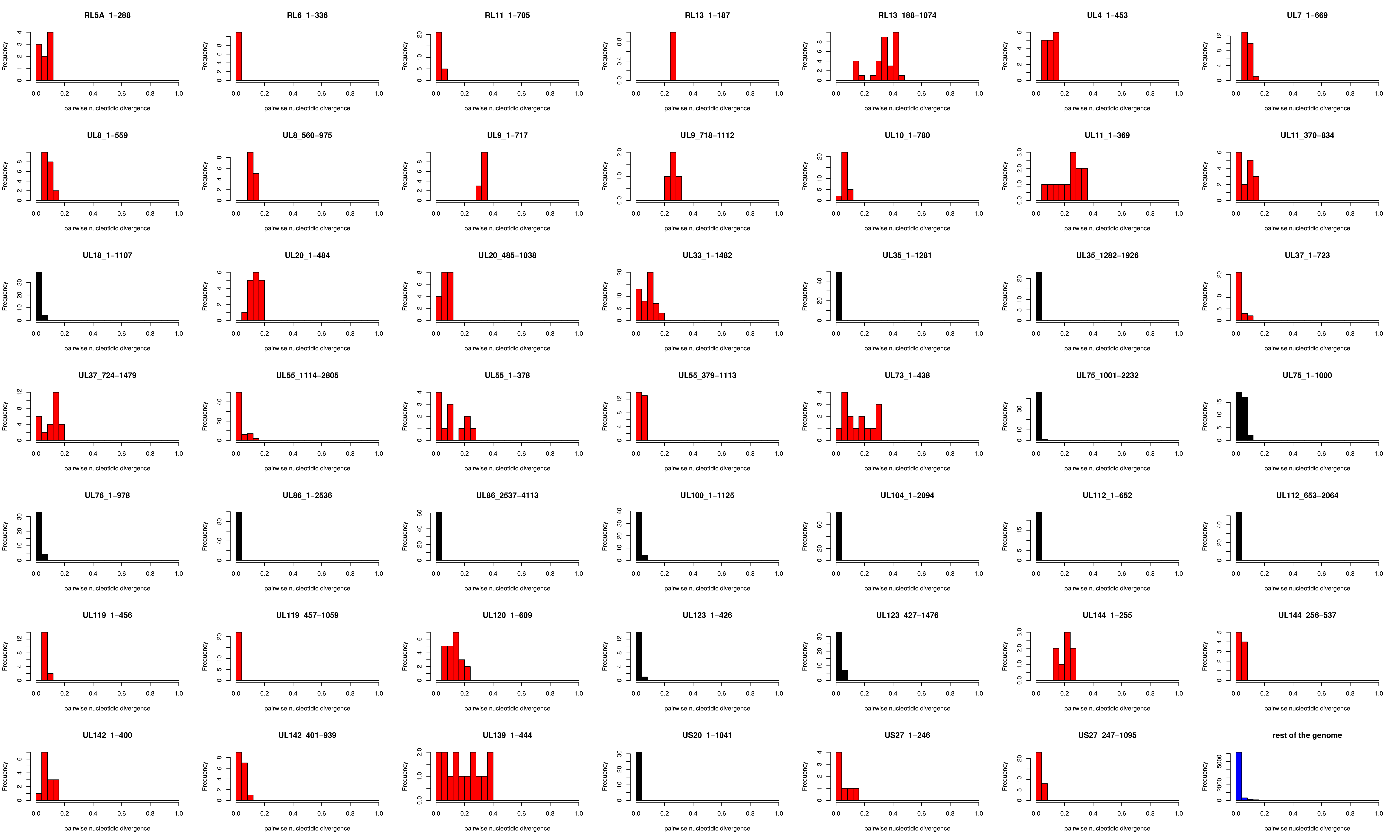

Supplement: Supplementary Data [file vew017_Supplementary_Data.zip › Sup_Figs/Figure S11.tif]

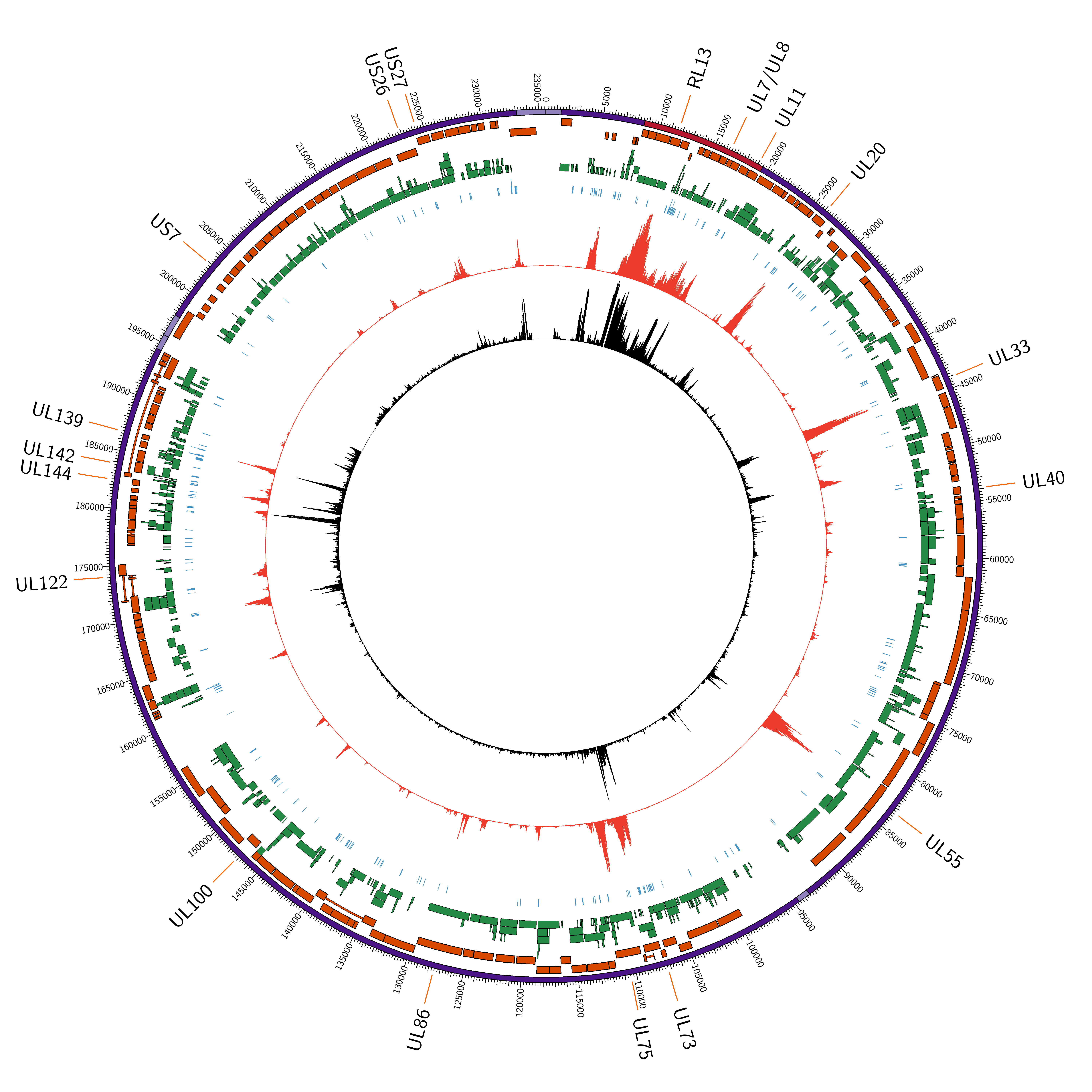

Supplement: Supplementary Data [file vew017_Supplementary_Data.zip › Sup_Figs/Figure S12.tif]

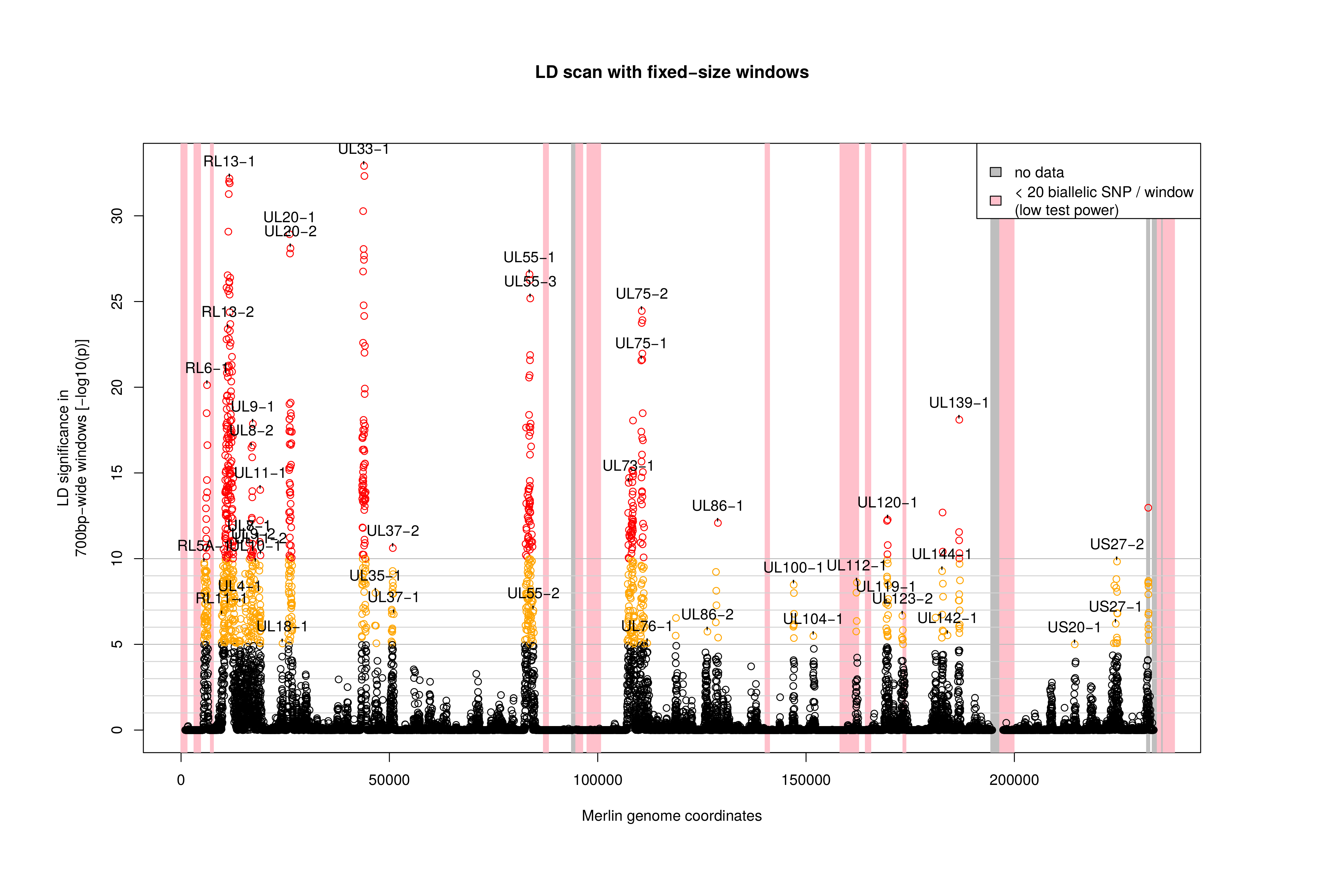

Supplement: Supplementary Data [file vew017_Supplementary_Data.zip › Sup_Figs/Figure S2.tif]

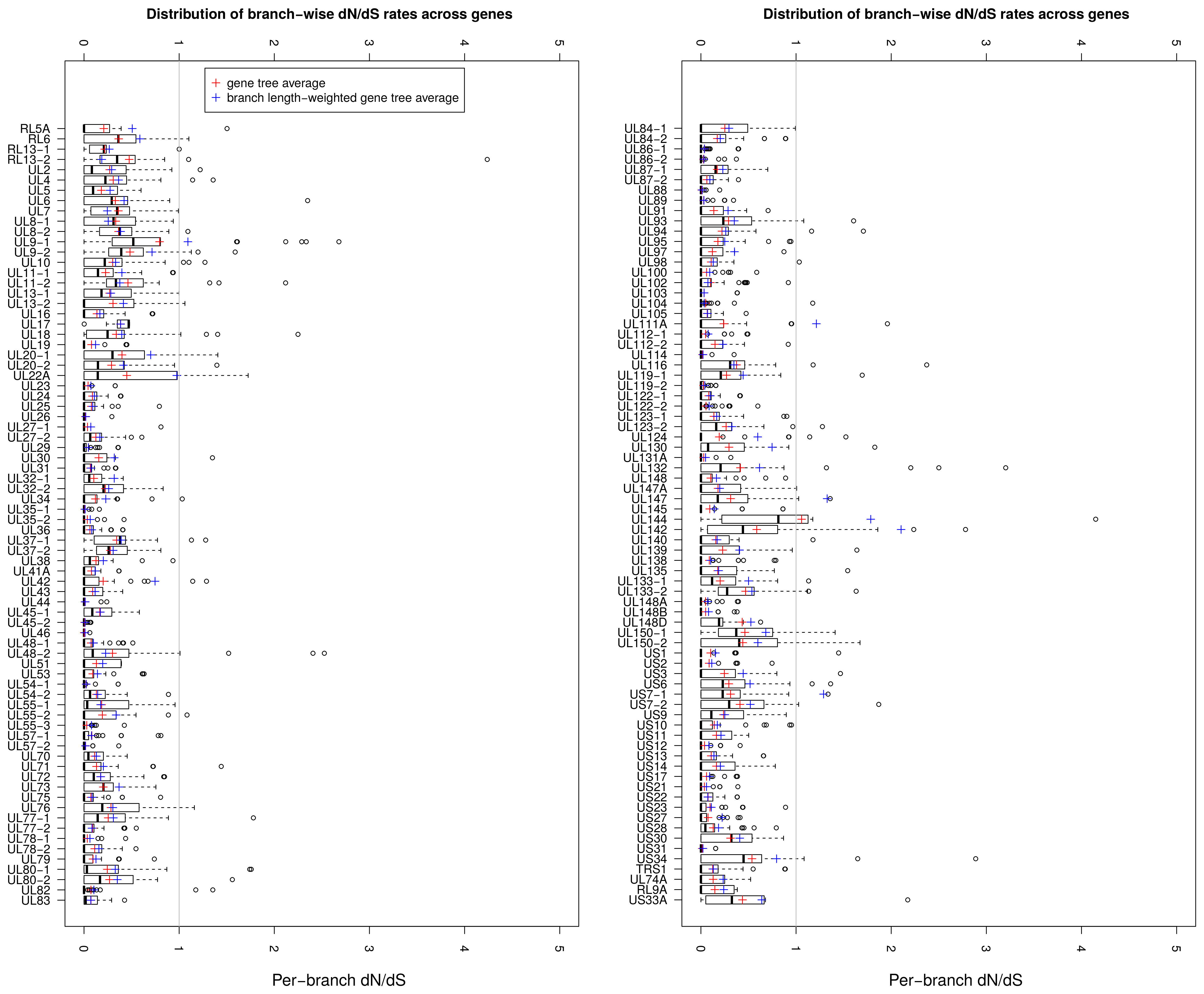

Supplement: Supplementary Data [file vew017_Supplementary_Data.zip › Sup_Figs/Figure S3.tif]

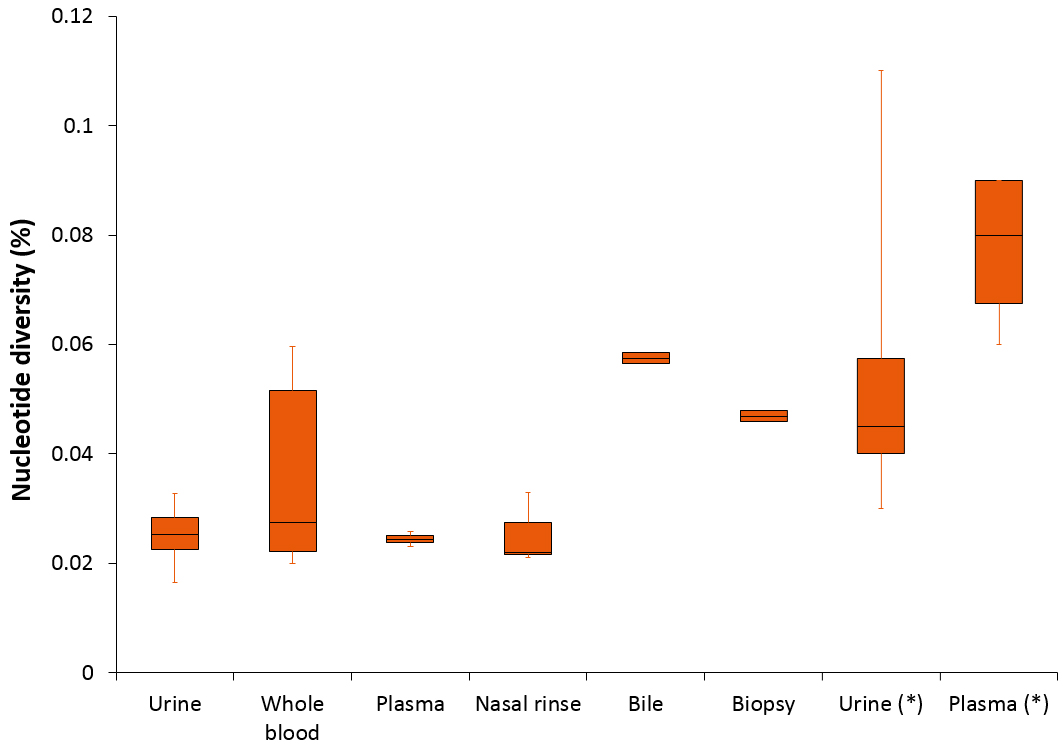

Supplement: Supplementary Data [file vew017_Supplementary_Data.zip › Sup_Figs/Figure S4.tif]

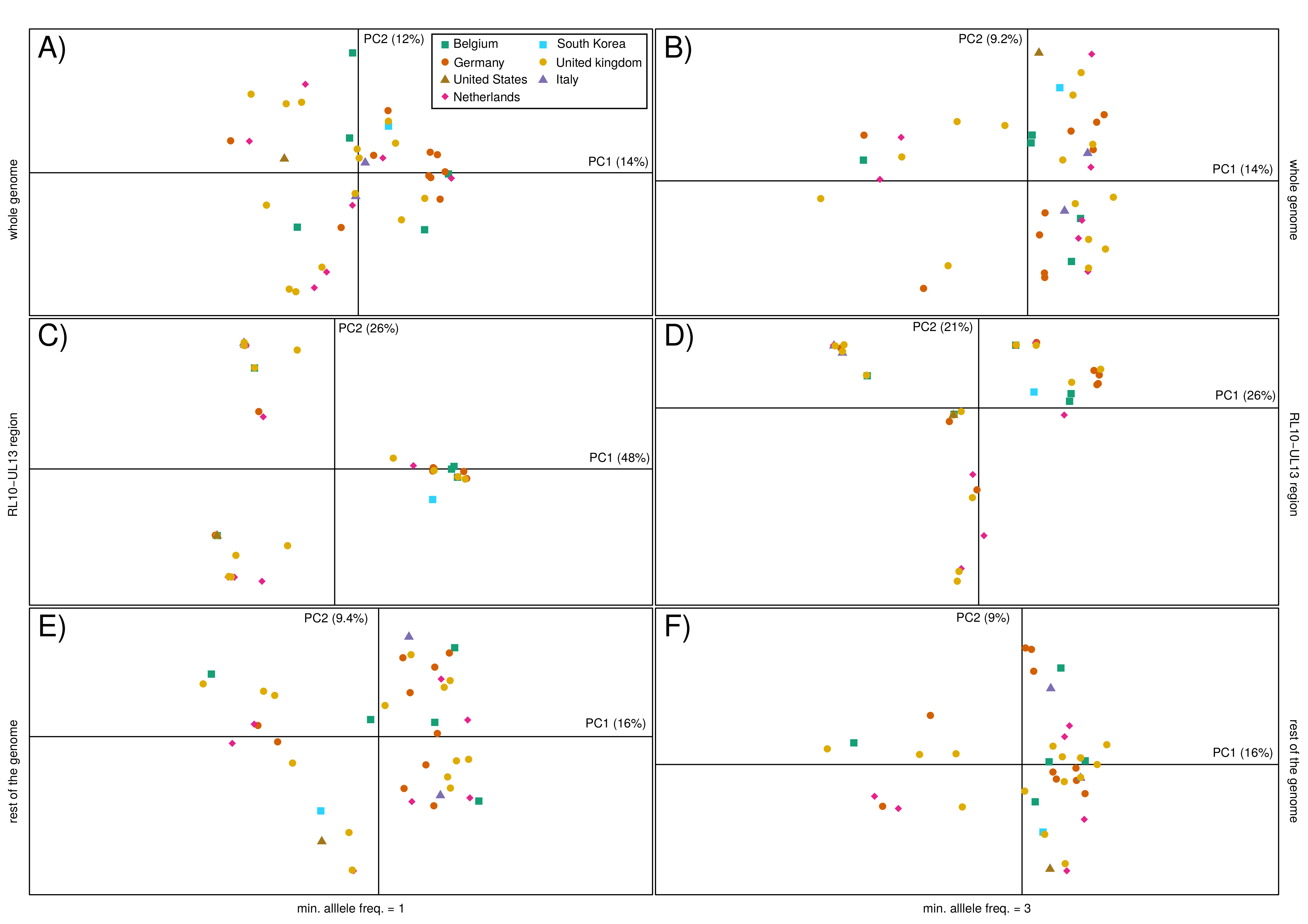

Supplement: Supplementary Data [file vew017_Supplementary_Data.zip › Sup_Figs/Figure S5.tif]

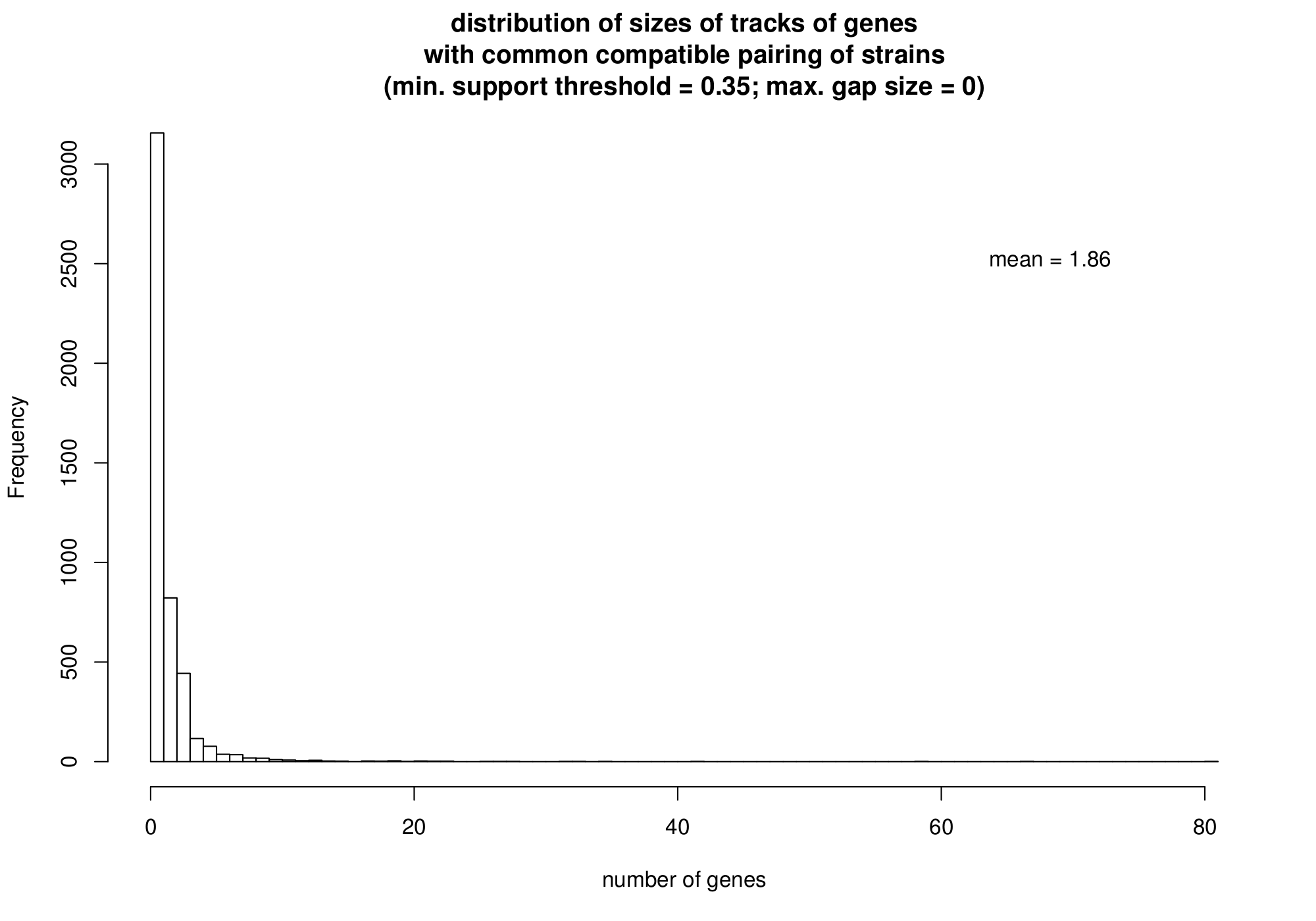

Supplement: Supplementary Data [file vew017_Supplementary_Data.zip › Sup_Figs/Figure S7.tif]

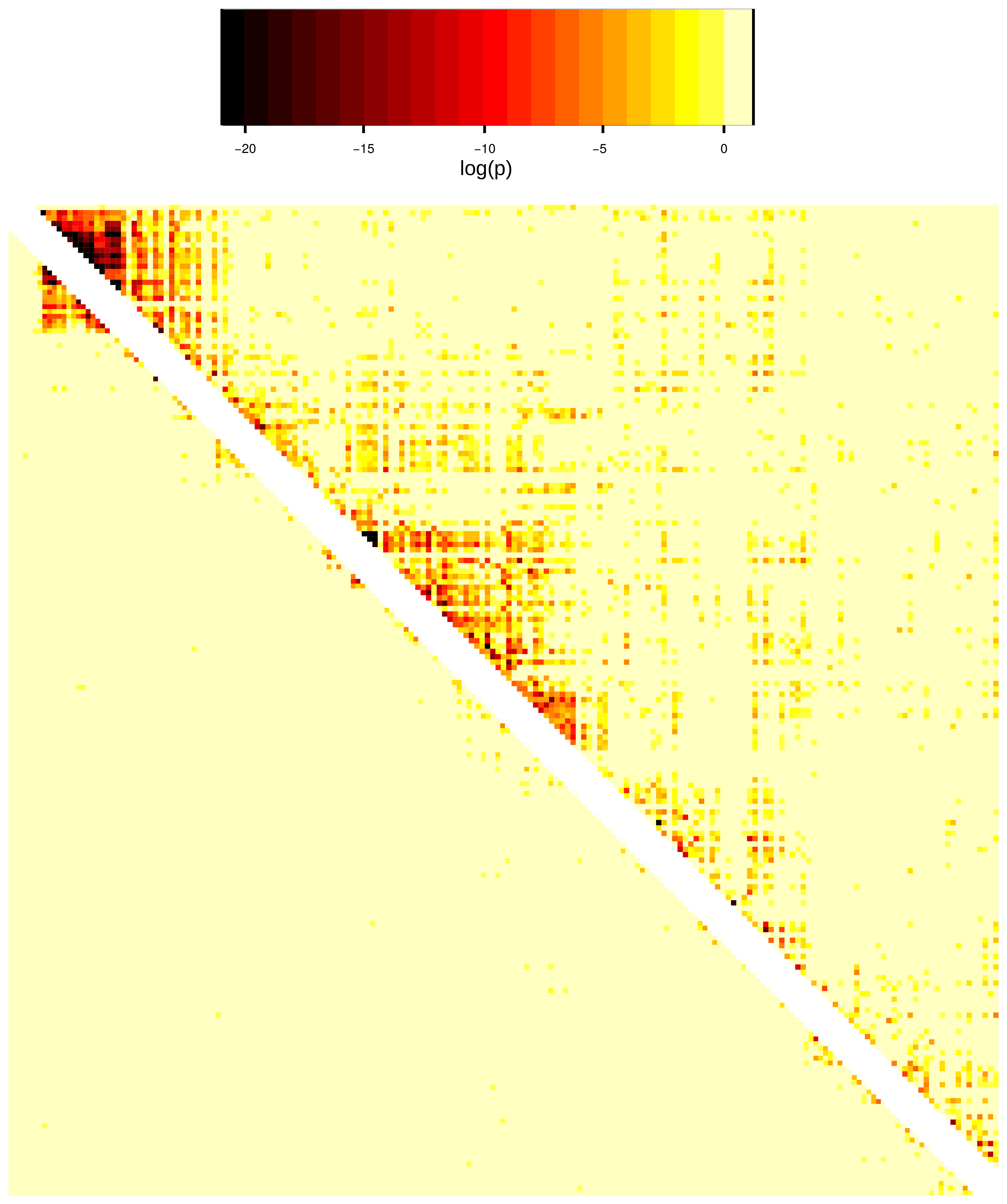

Supplement: Supplementary Data [file vew017_Supplementary_Data.zip › Sup_Figs/Figure S9.tif]

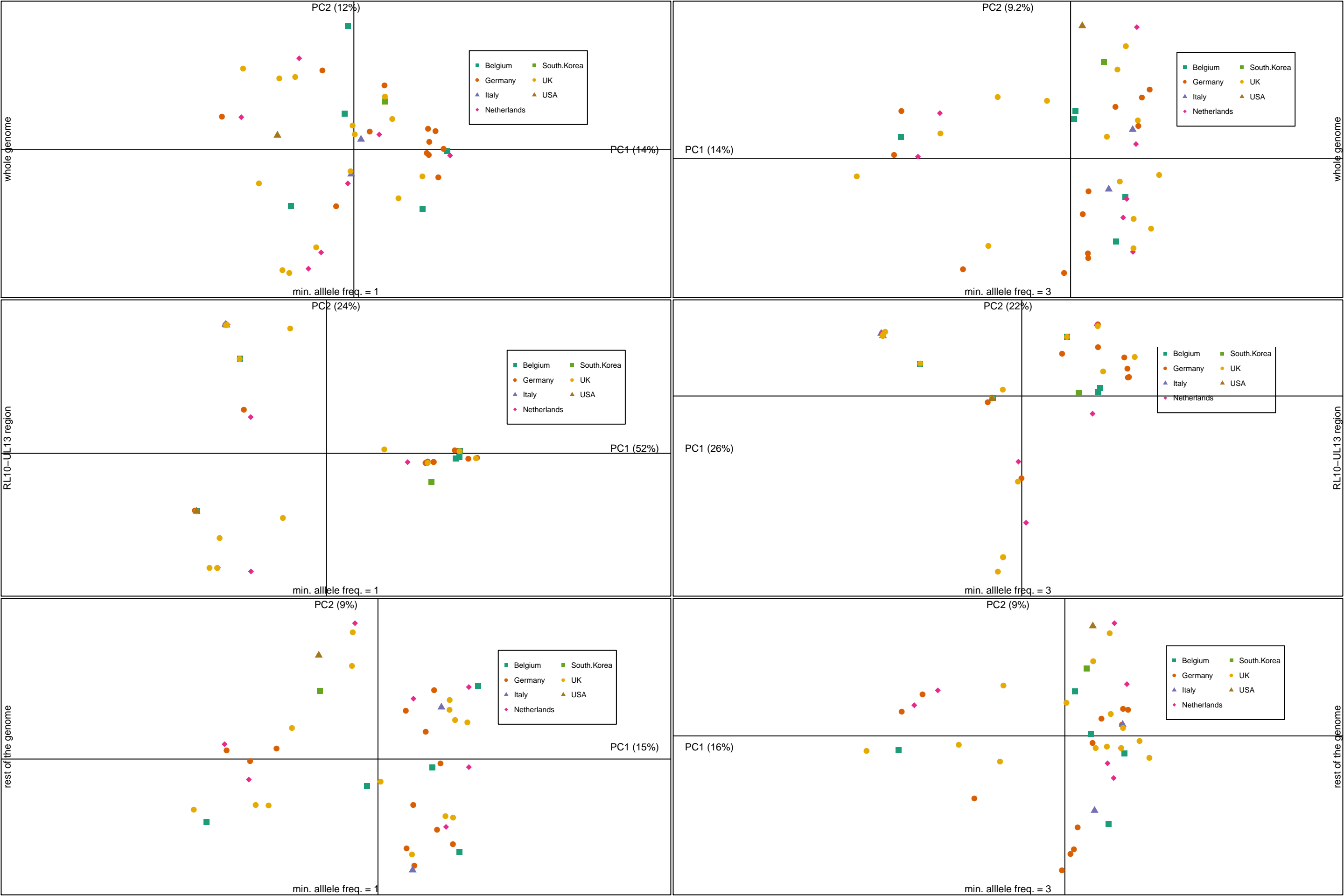

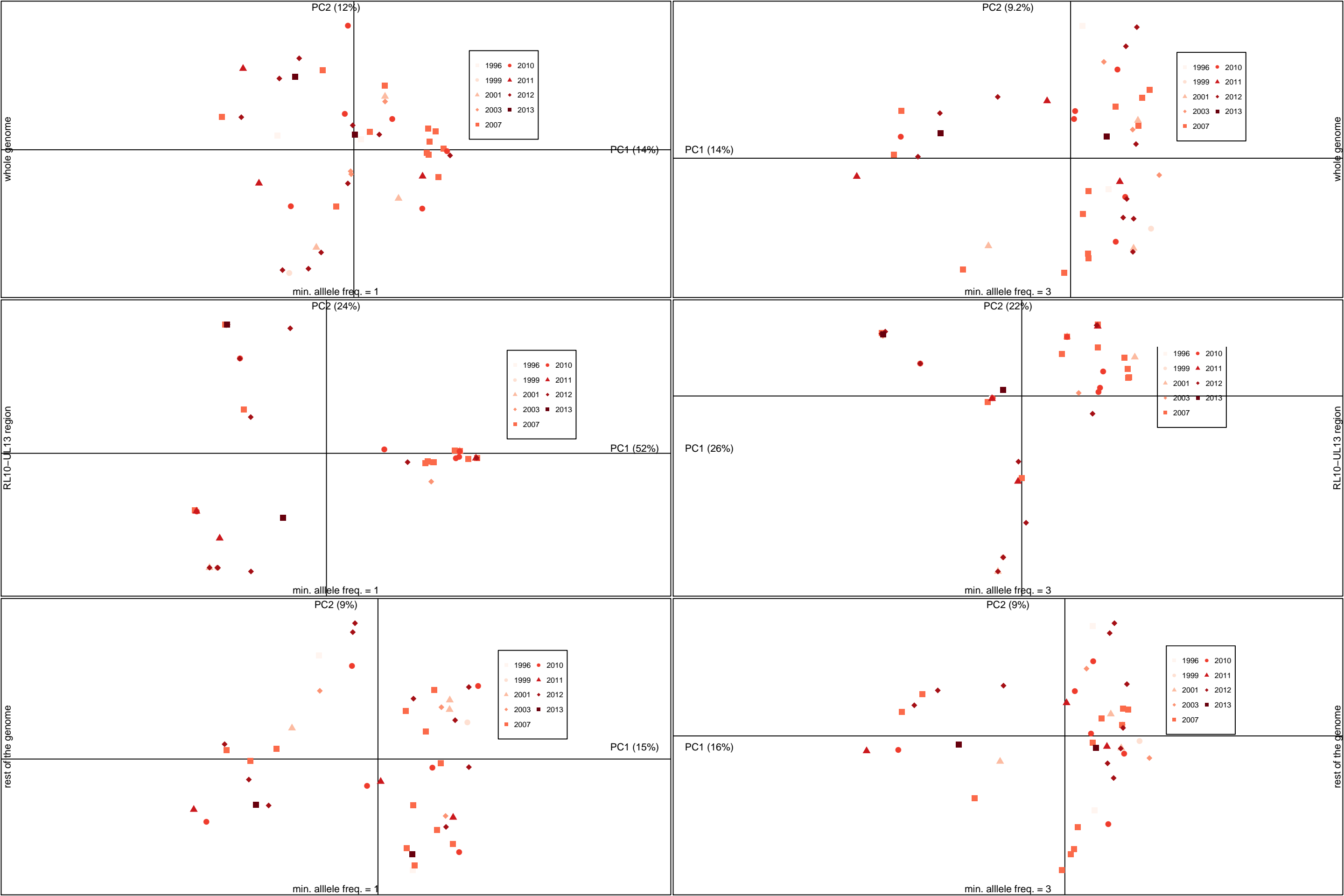

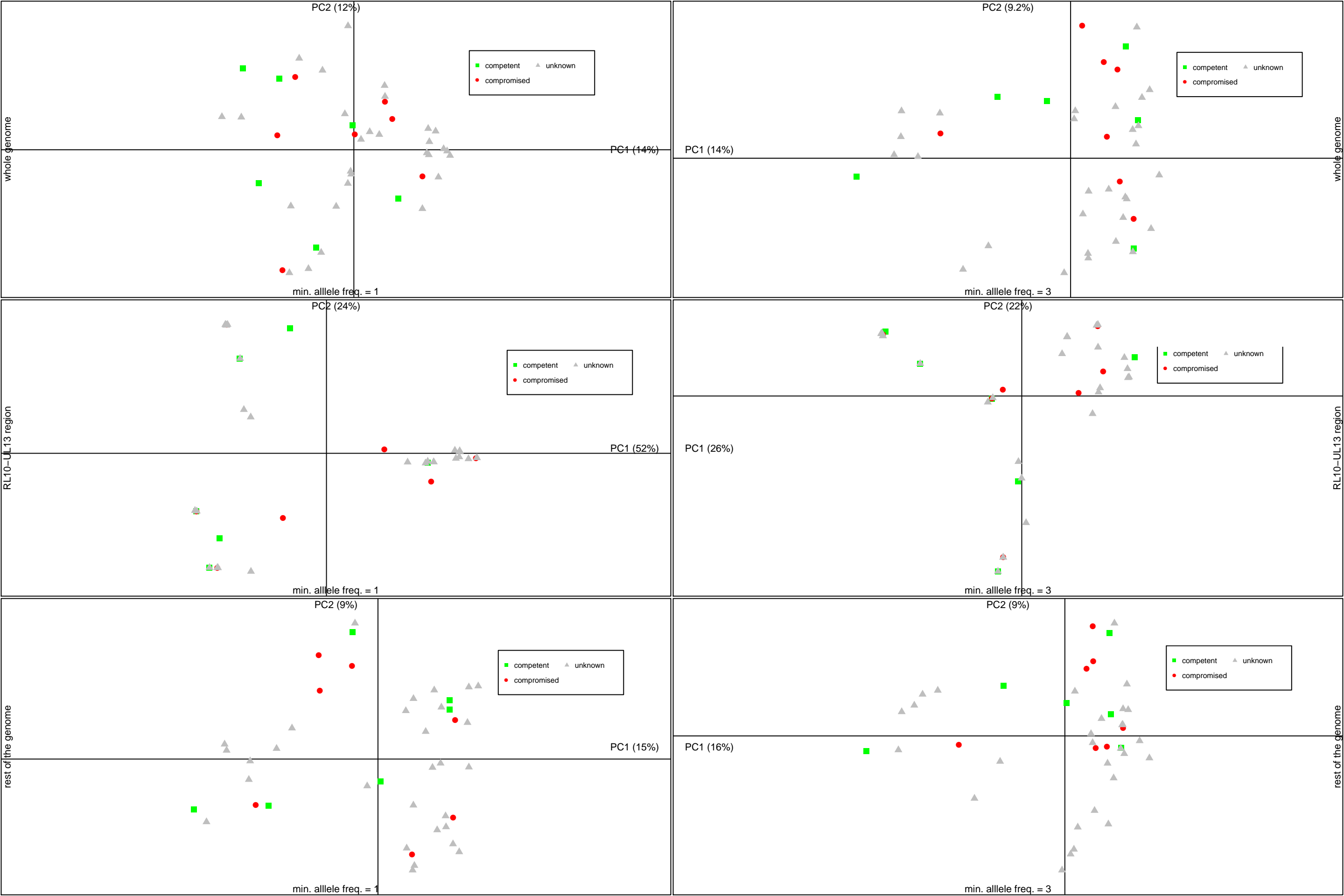

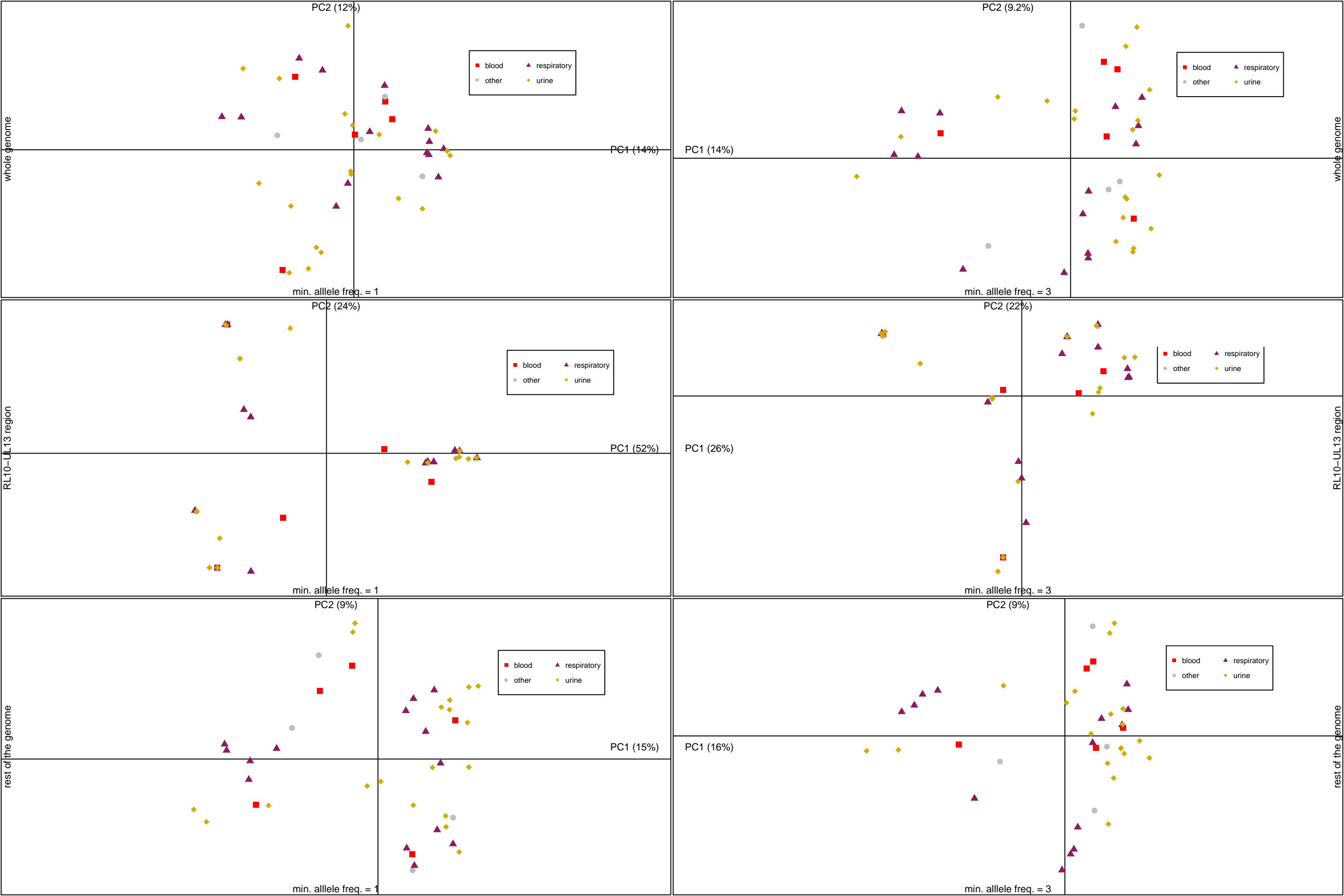

Supplement: Supplementary Data [file vew017_Supplementary_Data.zip › Sup_Files/SupFileS2.pdf]

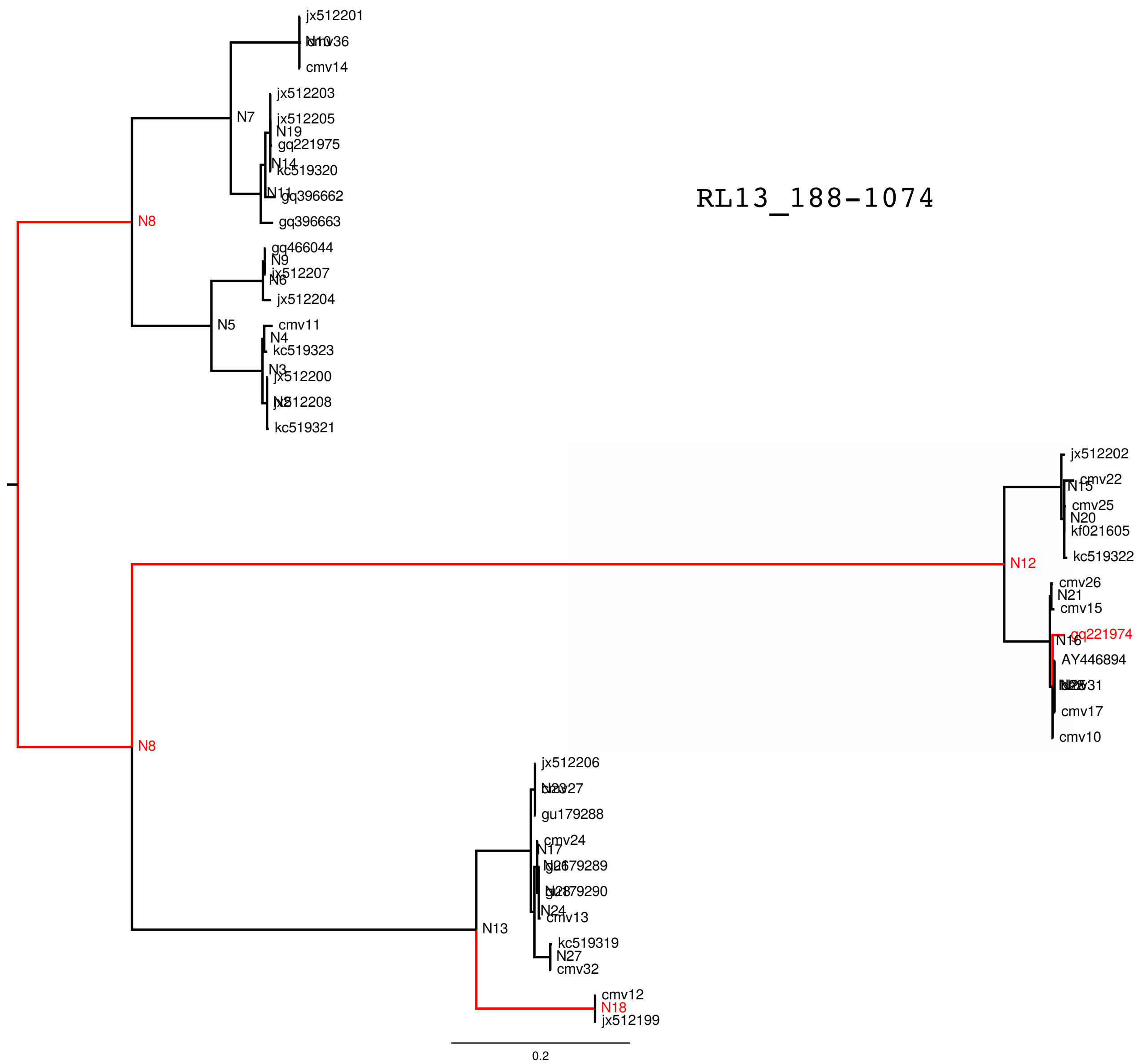

UL9\_1-717

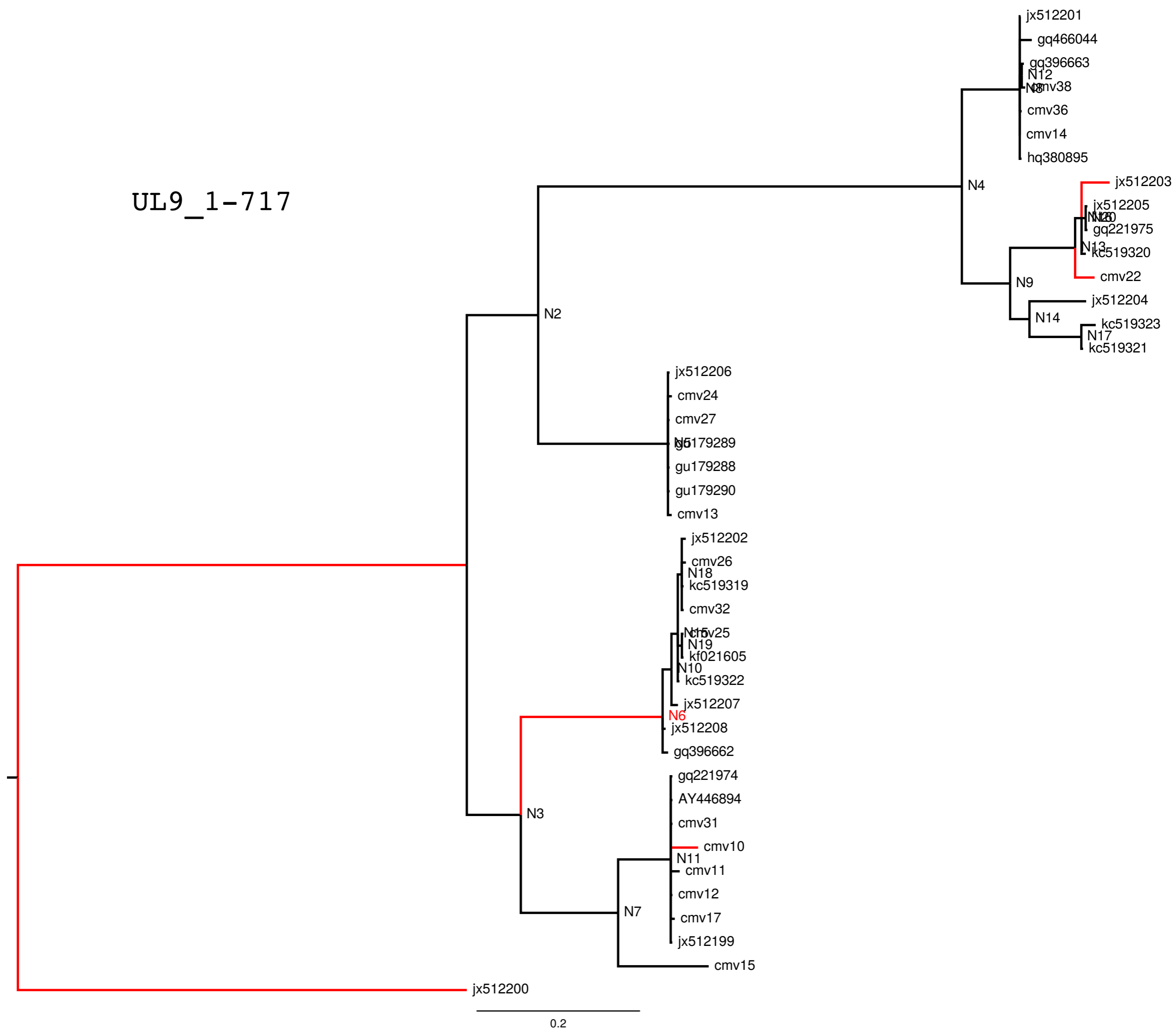

UL9\_718-1112

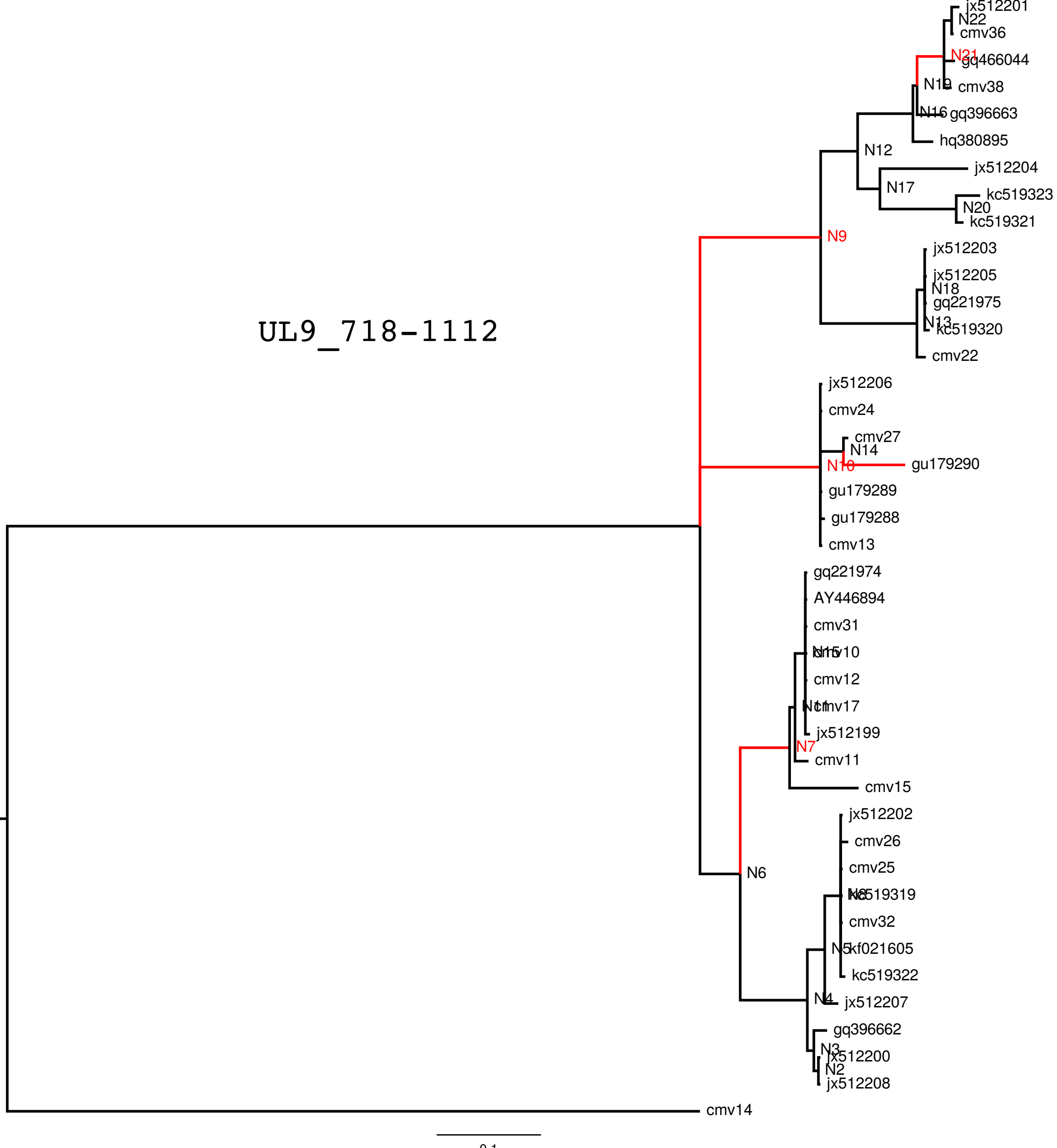

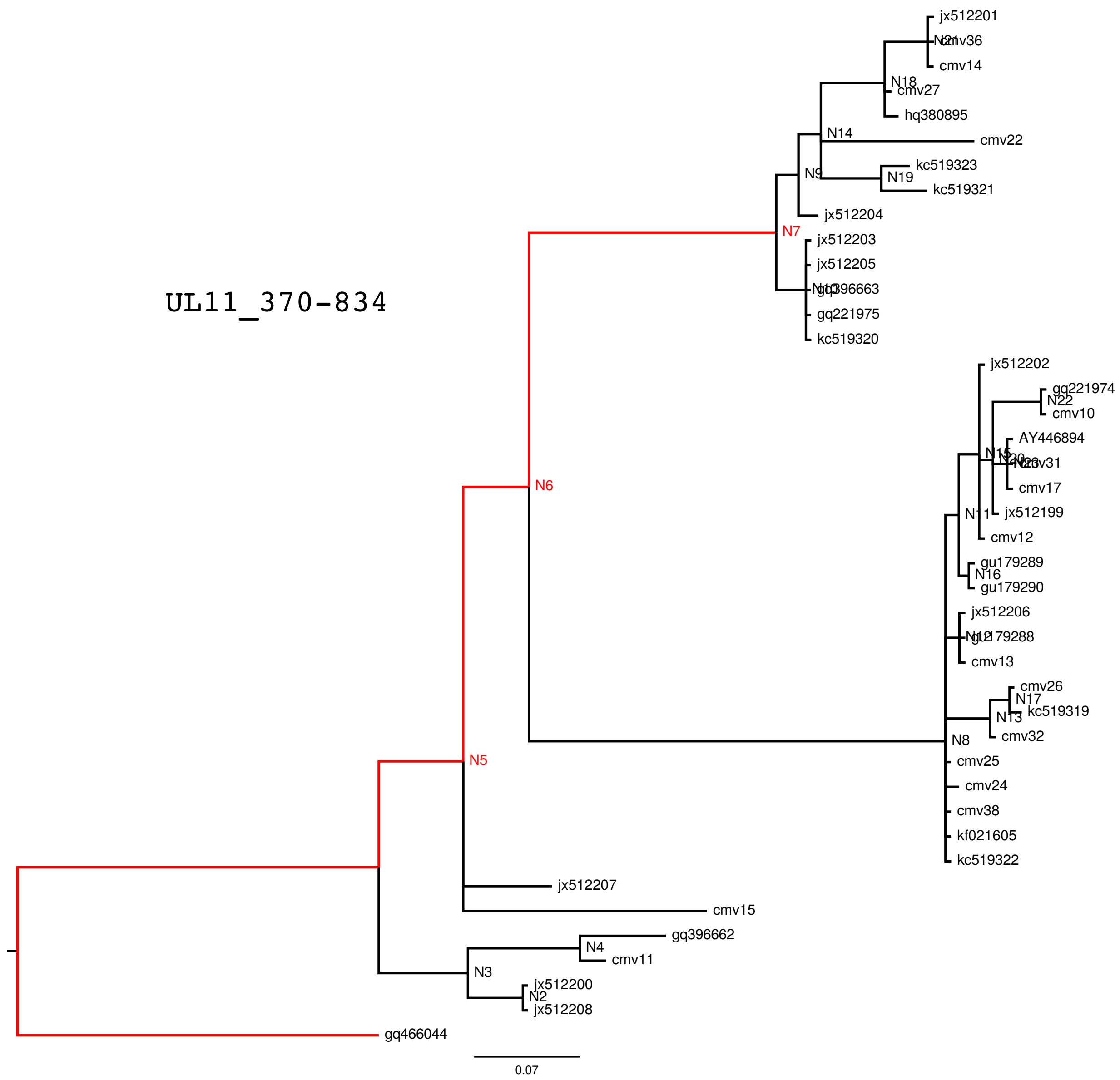

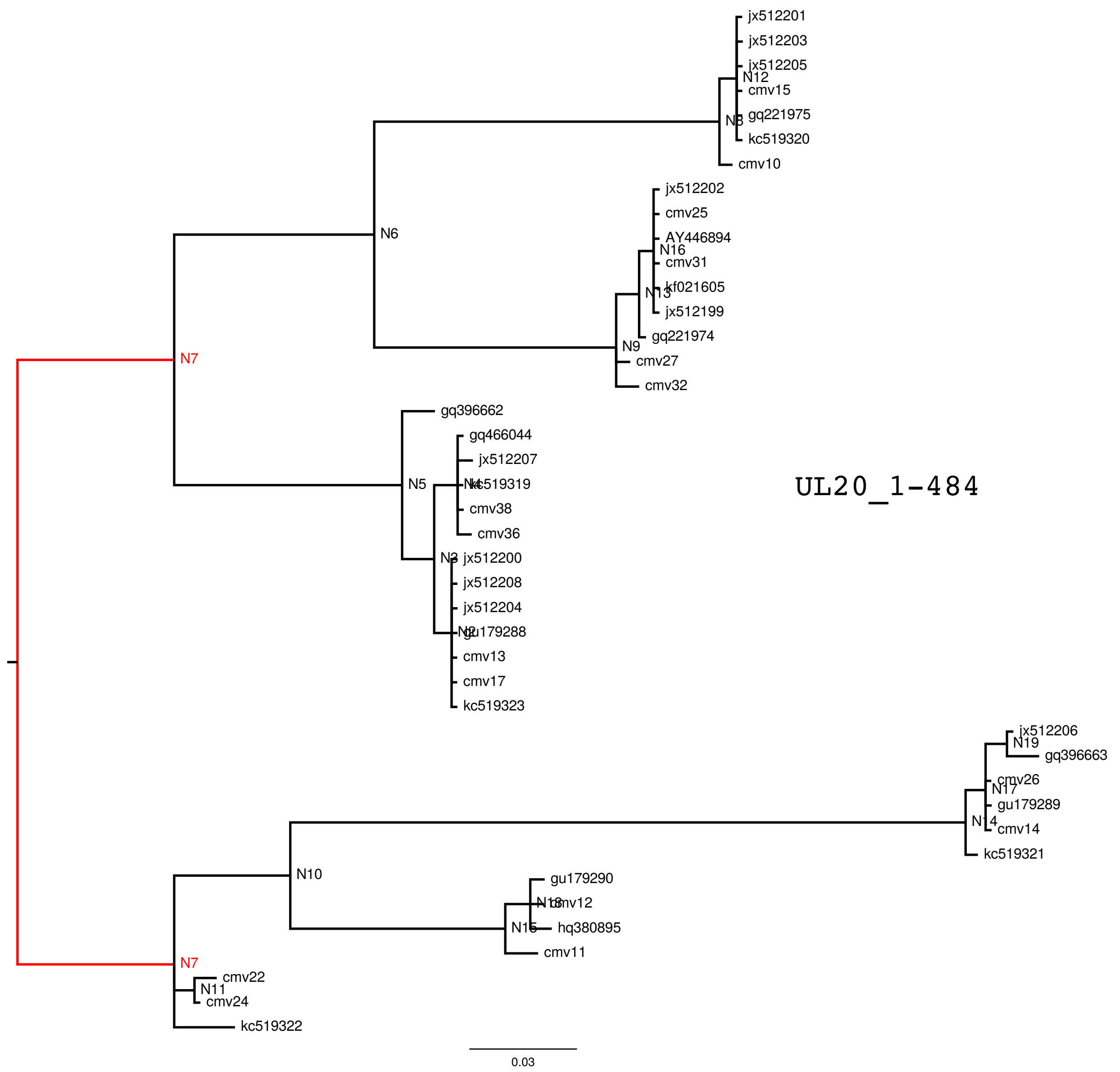

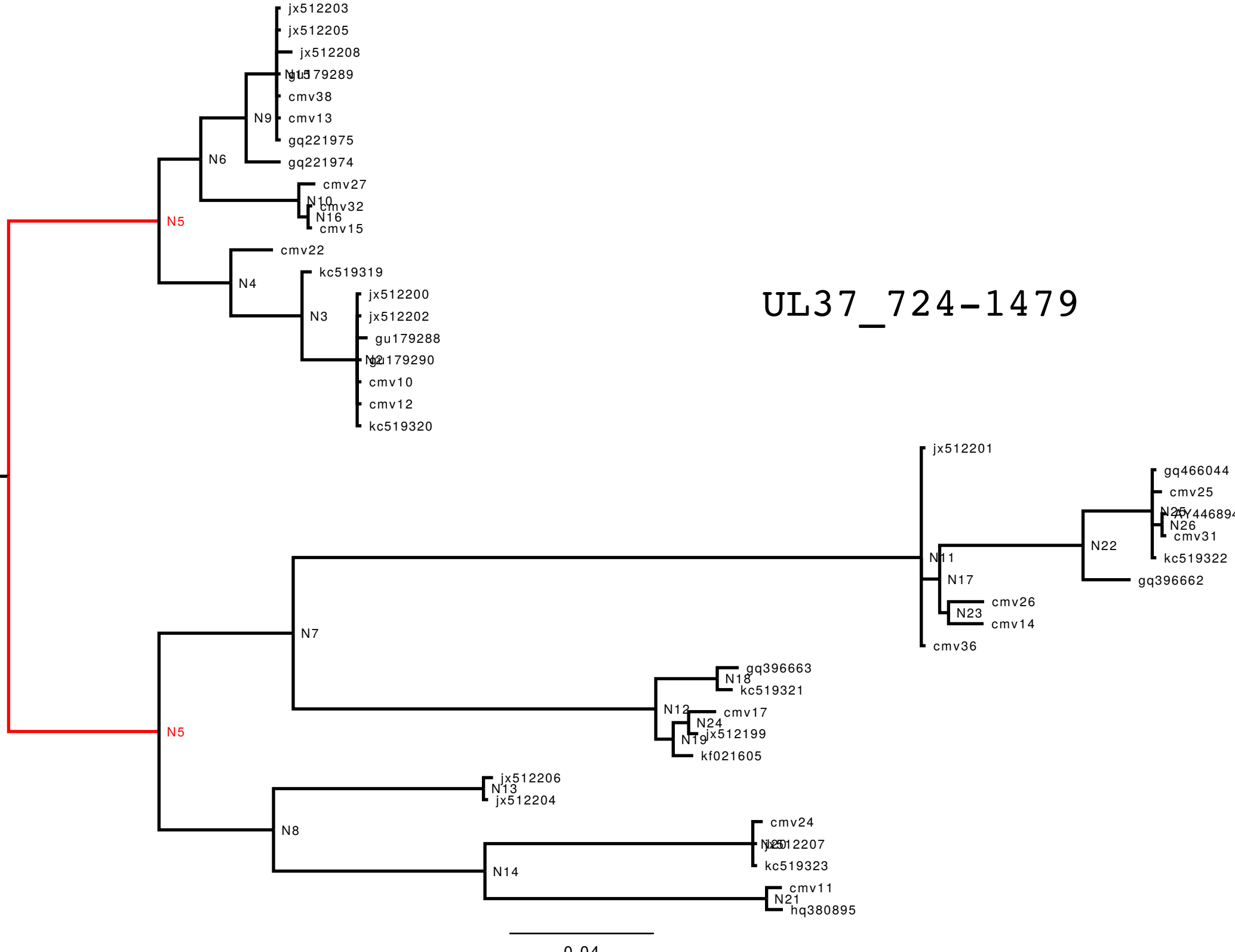

UL42\_1-387

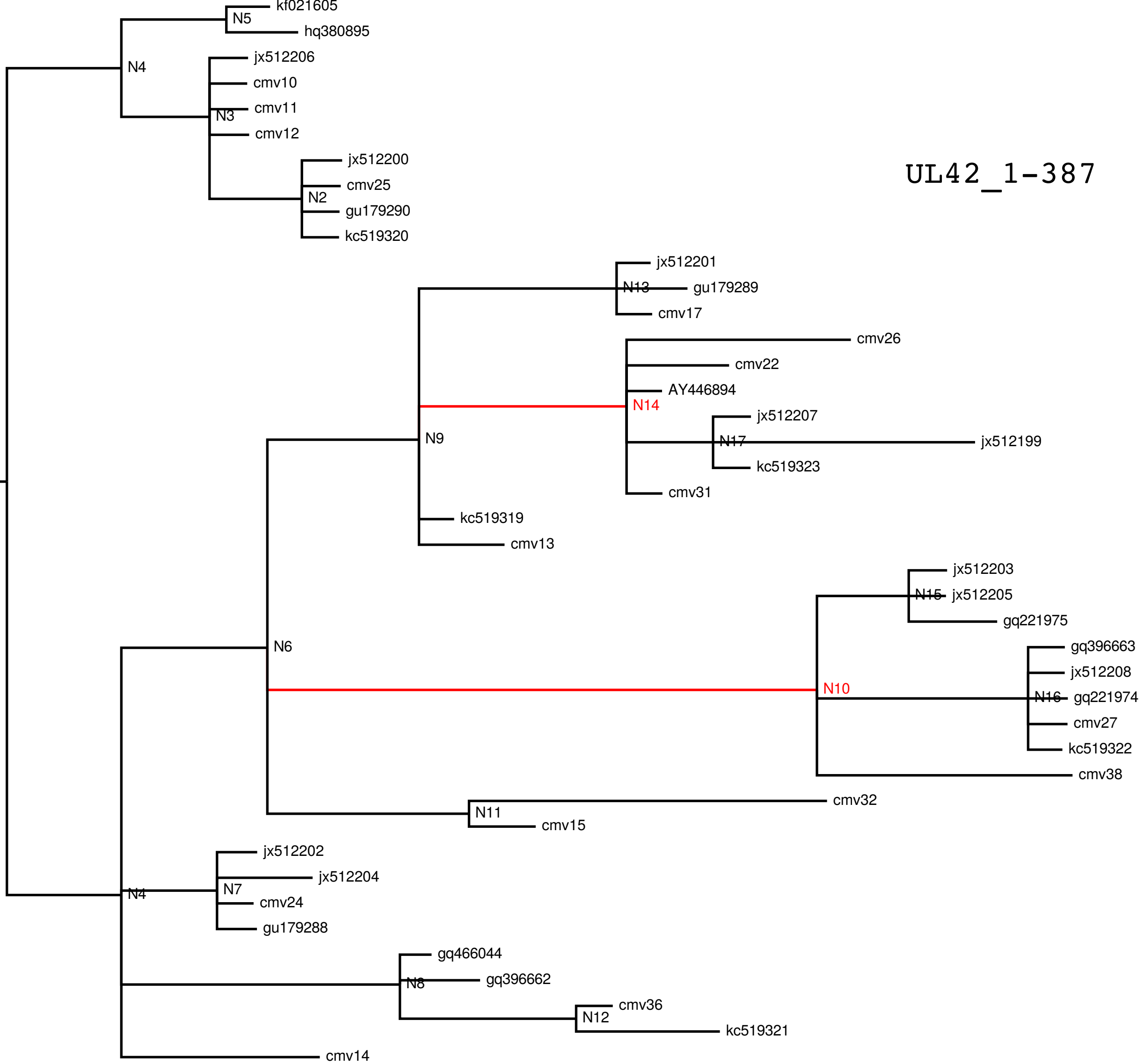

0.1

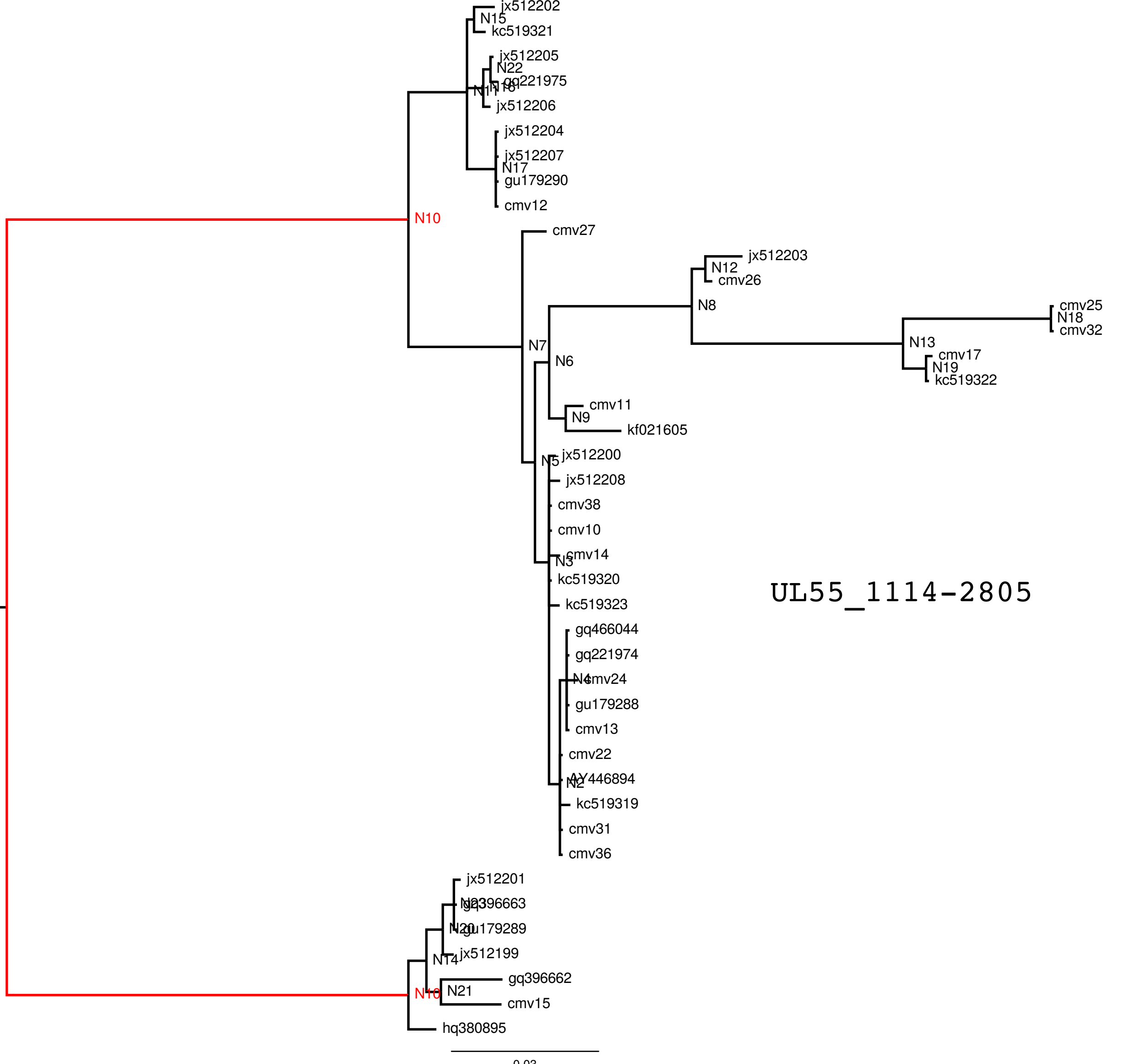

UL55\_1-378

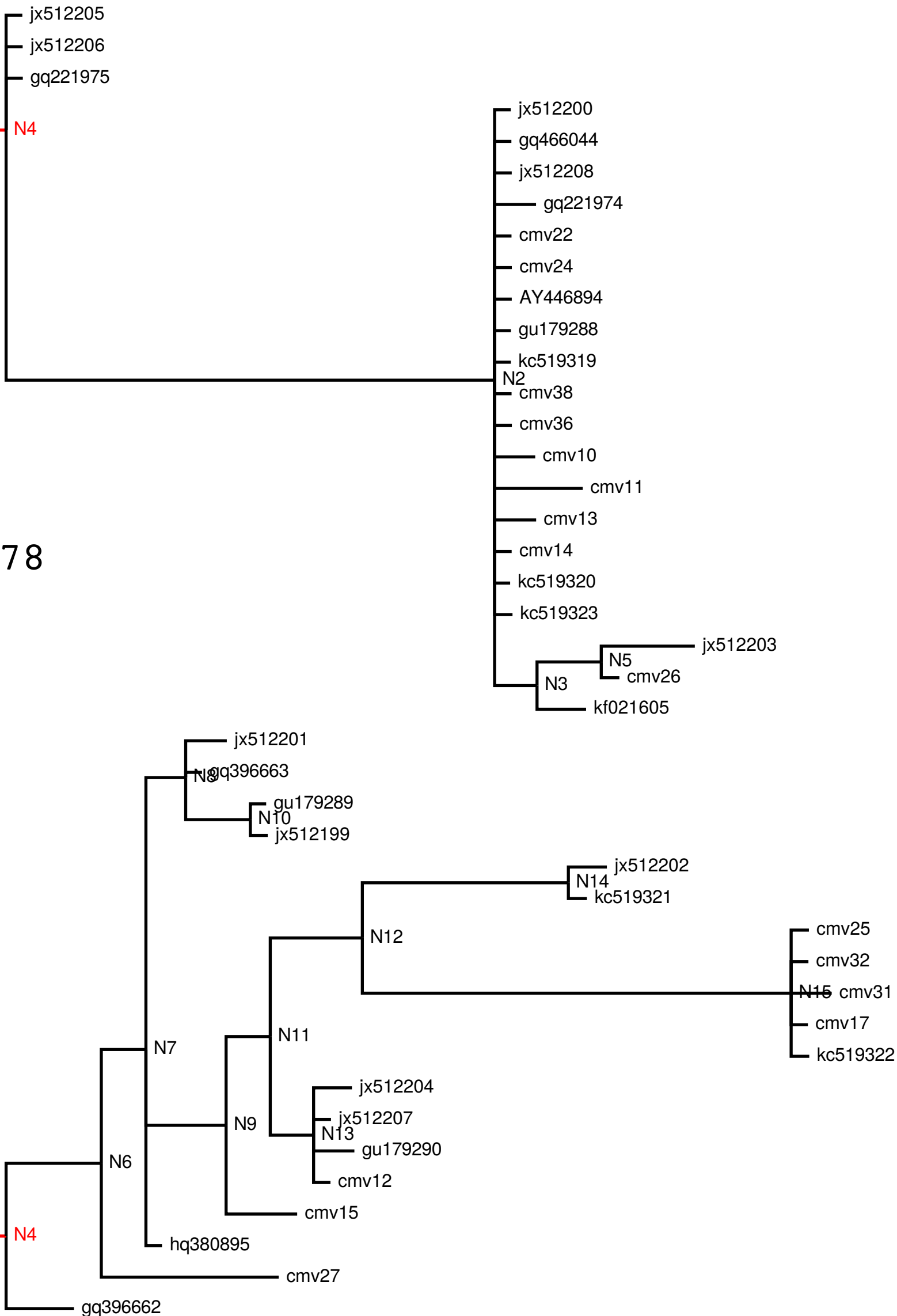

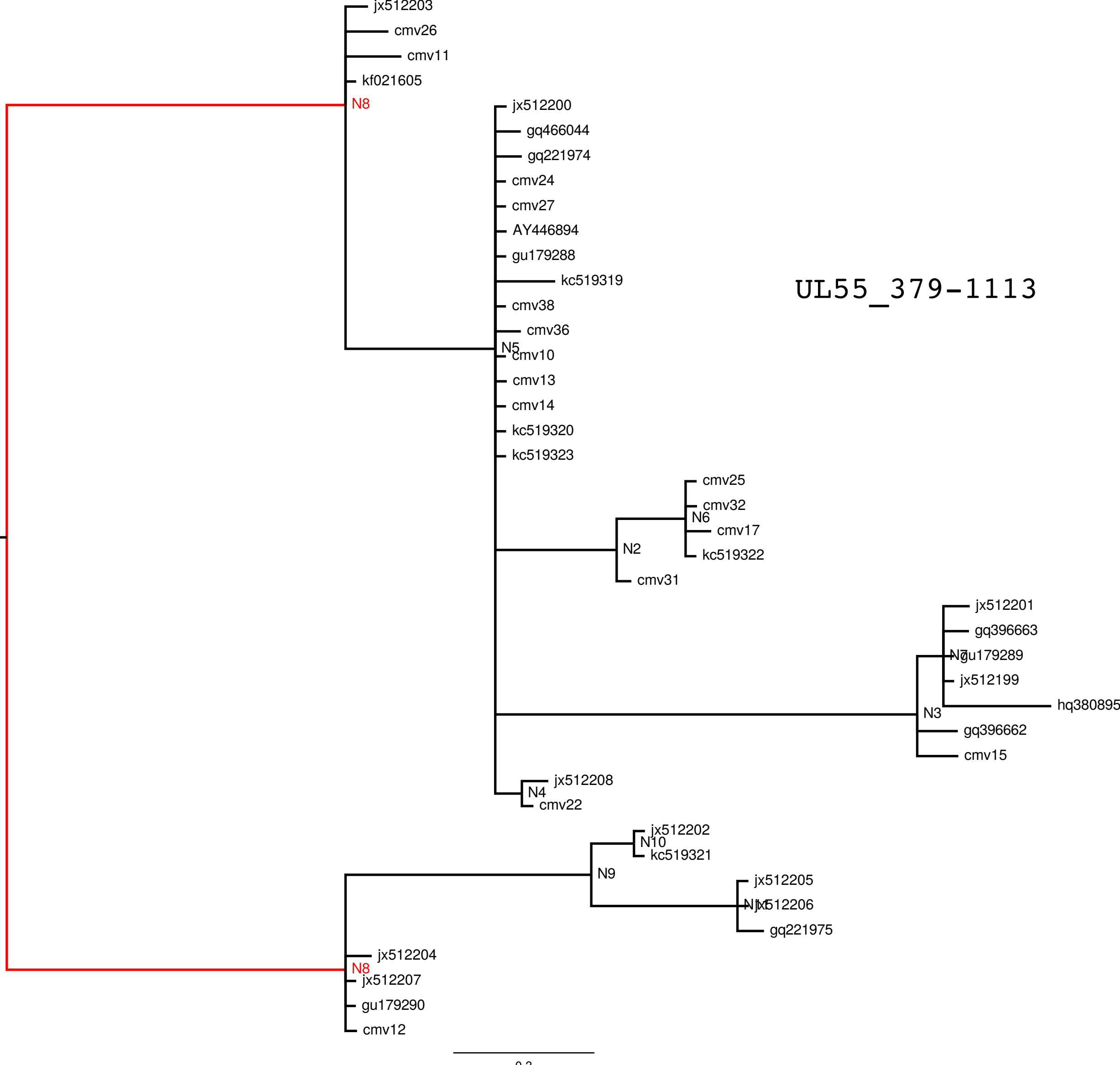

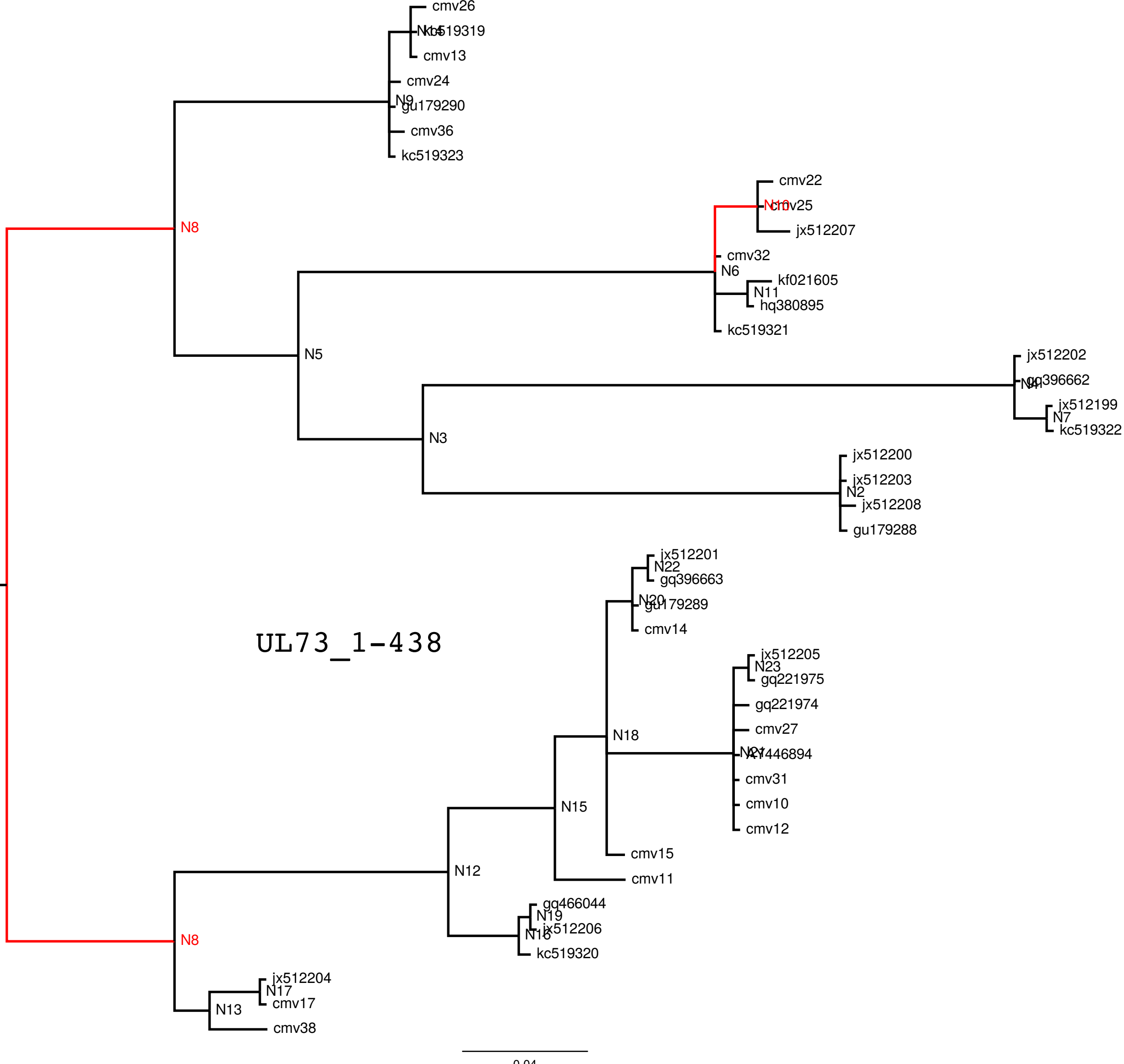

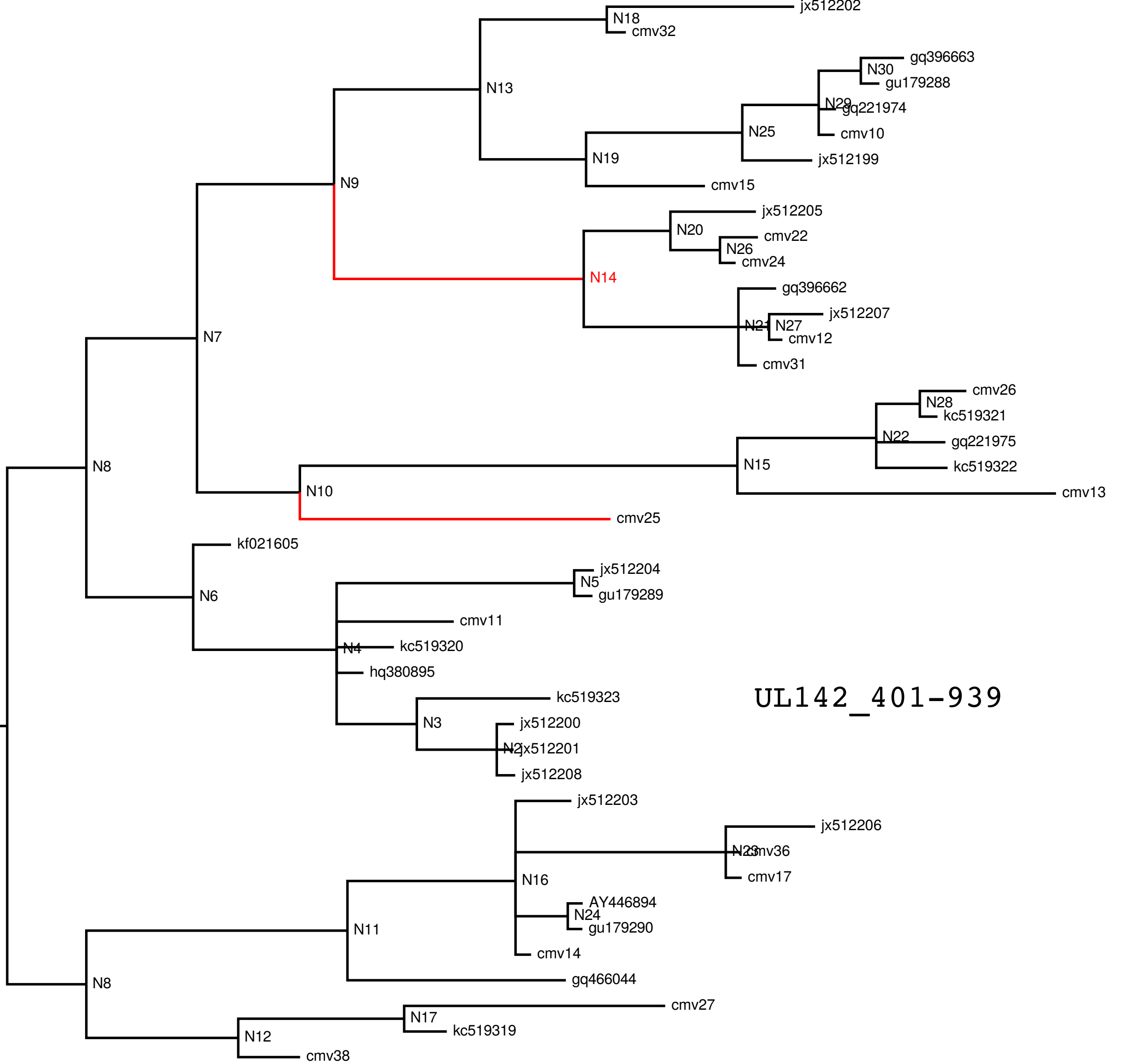

0.07

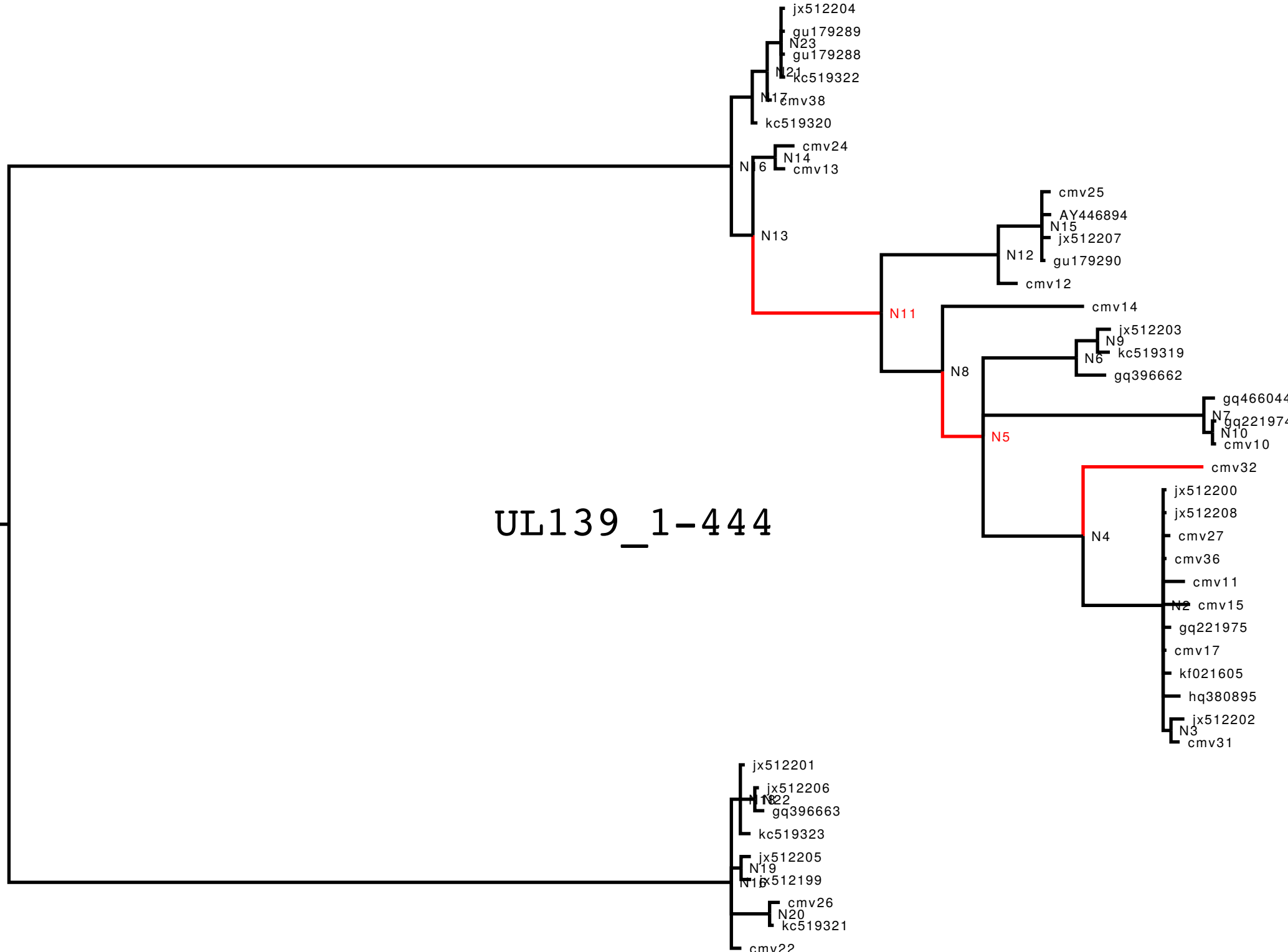

UL139\_1-444

0.08

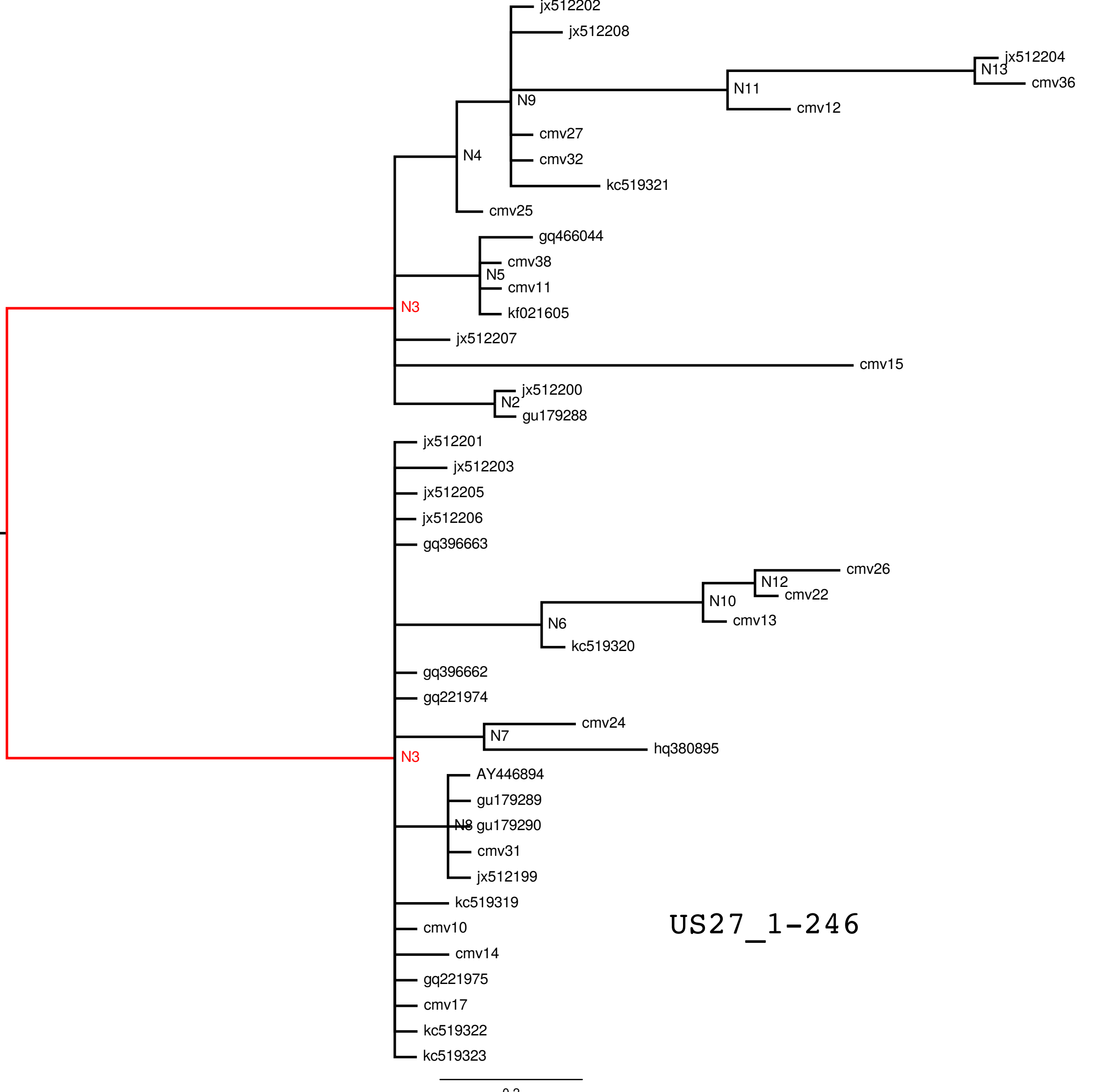

Supplement: Supplementary Data [file vew017_Supplementary_Data.zip › Sup_Files/SupFileS5.pdf]
